# Supplementary material for: Emergent collective behavior evolves more rapidly than individual behavior among acorn ant species
Source: Proc Natl Acad Sci U S A. 2024 Nov 22;121(48):e2420078121. doi: 10.1073/pnas.2420078121 (PMC11621464; doi:10.1073/pnas.2420078121)
Supplement: Supplementary file 1 — Appendix 01 (PDF) [file pnas.2420078121.sapp.pdf]

PNAS

Supporting Information for
Emergent collective behavior evolves more rapidly than individual behavior among acorn ant species

Grant Navid Doering, Matthew M. Prebus, Sachin Suresh, Jordan N. Greer, Reilly Bowden, Timothy A. Linksvayer

corresponding author: Grant Navid Doering
Email: gdoering@asu.edu

This PDF file includes:

Supporting text
Figures S1 to S8
Legends for Movie S1
Legends for Datasets S1 to S14
SI References

Other supporting materials for this manuscript include the following:

Movie S1
Datasets S1 to S14

SI text

Colony size analysis

Because prior studies have found that group size can influence aspects of colony activity cycles (1, 2), we conducted a supplementary analysis of the effect of colony size on our activity metrics by looking at the four species with the greatest number of colonies in our dataset (*T. ambiguus*, *T. curvispinosus*, *T. rudis*, and *T. rugatulus*). For each species, we fit an LME model with rhythmicity or period as the response variable, colony size (i.e., approximate number of adult ants inside the nest) as the fixed effect, and colony ID as a random effect. Colony size was not correlated with either of our colony-level traits in any of the four species (Figure S2a, b; see Dataset S8). A PGLS analysis likewise found no correlation for colony-level period vs. colony size (PGLS: t-statistic = 1.23, p-value = 0.23) nor colony-level rhythmicity vs. colony size (PGLS: t-statistic = 1.90, p-value = 0.07).

Disassembly experiment

Because our tests of isolated individuals in the main experiment relied on only a subset of ants from a given colony, it is possible that colony-level ultradian behavioral activity rhythms are determined by the average behavior of a specific behavioral caste (e.g., nurse workers) or a small number of especially influential ants within the colony (e.g., keystone individuals) (3). We tested this by isolating every adult ant from a colony in separate arenas and measuring their activity rhythms. We compared the distribution of individual-level time series activity traits in a colony with the corresponding colony-level values, which we obtained using automated analysis of the recording of the full, intact colony taken two days prior to the disassembly experiment.

Our results support the hypothesis that group-level characteristics of ants' collective ultradian rhythms (specifically the colony-level rhythmicity trait) are strongly contingent on the social environment of individual ants, i.e. that colony-level rhythmicity is an emergent trait. The colony-level rhythmicity values for the *T. rudis* and *T. obturator* colonies fell well outside of the distributions of individual-level rhythmicity values of isolated workers from these two colonies (Figure S3). In *T. rudis*, 98% of the individual rhythmicity values were lower than the colony value, and 100% of the individual rhythmicity values were lower than the colony value in *T. obturator*. This is consistent with earlier research that did not sample every worker in a colony; isolated individuals are usually less rhythmic than colonies (1, 2, 4, 5), and larger groups of workers extracted from colonies tend to be more rhythmic than smaller groups (2). For the *L. crassipilis* colony, which had a lower rhythmicity score than the other two colonies, the colony-level rhythmicity fell within the range of the individual-level rhythmicity values. In the case of the ultradian period (the trait which did not show evidence for emergent evolution), we found that the colony-level periods of *T. obturator* and *T. rudis* were close to the mean value of isolated individuals.

Small-nest worker experiment

Our main experiment exclusively used workers for the individual-level recordings that were foragers inside the nest box (except for the two *Leptothorax* individuals mentioned in the

Miscellaneous methodological details section below). The isolated ants in the main experiment were also filmed in 1.5ml microcentrifuge tubes that differed in shape from the artificial nests that full colonies were housed in. To ensure that neither the use of foragers nor the use of a particular filming environment for individuals influenced our interpretation of the results from the main experiment, we conducted an additional experiment (i.e., our small-nest experiment) using more isolated workers from nine species. These workers were isolated in small nests either alone or with a larvae item (Figure S5). The small nests consisted of a 1.5mm thick chipboard slat (22.5x22.5 mm) sandwiched between two plastic microscope slide cover slips. A 16x16 mm hole cut in the slat served as the nesting cavity, and a 1.5mm nest entrance was also cut into the side of the nests. To accommodate the larger size of the *Myrmica punctiventris* ants, the small nests given to these workers were thicker (3mm thick). Each nest was placed in the center of 60mm petri dish arenas, and the activity of each isolated worker in these arenas was tracked using optical flow. We included a cotton-plugged microcentrifuge tube filled with water in each petri dish to prevent workers from desiccating during the experiment. The colonies and isolated individuals used in the small-nest experiment were both filmed in a different room as the colonies and isolated individuals filmed in the main experiment. To reduce noise in the optical flow tracking in this new filming room, we filtered the individual-level time series by partitioning the image sequence frames into a regular grid and set any time points where activity was detected in more than ten regions of the frame to a value of NaN. Because the activity of an ant in a frame would generally only cause optical flow activity in one to two grid regions due to the individuals' small size, instances of optical flow activity in more than ten regions indicated noise in the tracking caused by slight changes in lighting. There were very few time-series data points that needed to be set to NaN because of noise; out of 374,400 total time-series data points in the new experiment, there were only 1,715 data points (i.e., 0.46% of the data) that needed to be set to NaN.

The rates of evolution for individual-level trait data in the small-nest experiment were estimated in the same way as described for the main experiment in the methods section of the main text. We assessed the statistical significance in the difference in evolutionary rates between individual-level and colony-level traits using the same bootstrap resampling approach used for the main experiment. Because we only had one colony of each species in the small-nest experiment, we estimated the colony-level evolutionary rates for the small-nest experiment using the colony-level data from the main experiment but pruned to include just the 9 species that we collected individual-level data for in the small-nest experiment. This allowed us to avoid any issues that might have been introduced by estimating rates using an asymmetric number of species across the individual and colony levels. Our disparity and phylomorphospace PGLS analyses for the small-nest experiment were performed in the same way as described for the main experiment.

The results of our small-nest experiment were qualitatively identical to the results of our main experiment. The estimated rates of evolution for colony-level rhythmicity were significantly higher than the estimated rates of evolution for individual-level rhythmicity in both the larvae treatments (Figure S6; $\sigma^2_{Colony-level}$ vs. $\sigma^2_{Individual-level}$ bootstrap p-value = 0.0001) and the no-larvae treatments of the small-nest experiment (Figure S6; $\sigma^2_{Colony-level}$ vs. $\sigma^2_{Individual-level}$ bootstrap p-value = 0.0001). The estimated rates of evolution for individual-level period was not significantly different than the estimated rates of evolution for colony-level

period in either the larvae treatments (Figure S6; $\sigma^2_{Colony-level}$ vs. $\sigma^2_{Individual-level}$ bootstrap p-value = 0.0963) or the no-larvae treatments of the small-nest experiment (Figure S6; $\sigma^2_{Colony-level}$ vs. $\sigma^2_{Individual-level}$ bootstrap p-value = 0.1033). The estimated total phenotypic diversity of colony-level behaviors was significantly greater than the phenotypic diversity of individual-level behaviors in both the larvae treatment (Figure S7b; colony-level disparity vs. individual-level disparity bootstrap p-value = 0.0001) and the no-larvae treatment (Figure S7c; colony-level disparity vs. individual-level disparity bootstrap p-value = 0.0001). Analogous collective/individual trait pairs were not evolutionarily correlated in either the larvae treatment (Figure S7d; PGLS: rhythmicity – t-statistic = 0.75, p-value = 0.48; period – t- statistic = 1.54, p-value = 0.17) or the no-larvae treatment (Figure S7e, PGLS: rhythmicity – t-statistic = 0.77, p-value = 0.46; period – t- statistic = -0.12, p-value = 0.91).

Miscellaneous methodological details

The optical flow method used for our disassembly experiment has been previously implemented for studying ant activity (6). Our use of optical flow relied on the Farneback technique. Greater details on this method can be found in other sources (see refs. (6, 7)), but the basic principle behind this technique is that changes in the brightness values of pixels in successive frames of an image sequence are used to estimate the motion of objects in the frames. An optical flow vector is calculated for each pixel in a pair of frames, and a given optical flow vector conveys the approximate direction and velocity (pixels/frame) of the object for that pixel. Larger average optical flow magnitudes can thus be used as a proxy for the proportion of ants in the nest that are active in the case of colony recordings or individual walking speed in the case of isolated individuals.

As mentioned in the Methods section of the main text, the *Myrmica punctiventris* colonies used in the main experiment required a larger nest cavity to accommodate their larger body size. The *M. punctiventris* colony used in the small-nest experiment was also kept in a nest with a larger cavity than our standard nest design (our standard design was 1.5 mm thick with a nesting cavity 29x44 mm in size). The nest of the *M. punctiventris* colony in the small nest experiment used a 3mm thick chipboard slat sandwiched between two microscope slides with a rectangular nesting cavity (39.5x54.5 mm). Lastly, due to a shortage of our standard nest designs, two of the colonies in the small-nest experiment (colony JRCT2410 and colony JR4T2406) and seven out of the 58 unique *T. ambiguus* colonies used in the main experiment needed to be housed in slightly thicker nests (2 mm thick) with a modestly smaller cavity (25x34 mm) compared to our standard nest design.

Five of the colonies analyzed in this study were chimeric. Specifically, two out of the 29 *Temnothorax curvispinosus* recorded for our study contained the dulotic social parasite *Temnothorax americanus* and another two of the colonies contained the workerless inquiline *Temnothorax minutissimus*. These colonies were classified as *T. curvispinosus* in our analyses since these parasites were only a small minority of each nest's population. Additionally, one of the colonies classified as *Leptothorax athabasca* contained a minority of workers and larvae of *Leptothorax calderoni*. The two *Leptothorax* AF-erg ants used for the individual-level recordings were taken from a small colony that was collected in 2021. At the time of our study, this colony had declined and only consisted of these two ants. The large *L.* AF-erg colony that was used for

our colony-level activity recordings did not have any foragers in their nestbox on the occasions of our individual-level recording sessions, so we resorted to using the two *L*. AF-erg individuals from this other colony fragment for our individual-level assays.

In our recordings of isolated individuals, ants would occasionally fail to be detected by our tracking code. We therefore only included individual-level time series in our analysis if the focal individual was detected in at least 70% of the frames from the recording. Because instances of failed detection generally occur when the ant is resting and occluded by part of the observation arena, we followed the protocol used in previous work and assigned a value of zero prior to the missing data-points in the activity time series that were used for the analyses prior to the application of the Gaussian smoothing (4).

We took a colony-level recording of a *Temnothorax emmae* nest, but workers of this colony appeared injured, and no workers were used in individual-level tests, so we did not include it in our study. A single worker of *Temnothorax wardi* was also recorded during our individual-level tests, but we did not have a colony to record (only three workers), so we could not include this species in our analyses. Also, tracking issues prevented the velocity data from one *Leptothorax athabasca* worker from being used in the open field velocity analysis, so there were 576-2=574 total workers whose velocity data was analyzed from the open field assay. There were tracking issues with one of the recordings of colony WRT2 (a *T. nitens* colony) and one of the recordings of colony BFLT2 (*T. obturator*), so these two colony-level recordings needed to be excluded. Two of the *Leptothorax athabasca* individuals recorded for the open field assay were pseudogynes, which are unmated queens that behave like workers (8). Pseudogynes are not uncommon in colonies of *Leptothorax* (9).

In our disassembly experiment, a small proportion of ants from the *Temnothorax rudis* colony as well as the *Temnothorax obturator* colony and the *Leptothorax crassipilis* colony escaped their arenas during filming and needed to be returned to their respective nestboxes. Five workers escaped (out of 70 ants total) in the *T. rudis* recordings, one worker escaped (out of 18 ants total) in the *T. obturator* recordings, and one worker escaped (out of 16 ants total) in the *L. crassipilis* recordings. Similarly, eight isolated worker ants (five *T. nitens* workers, two *T. rudis* workers, and one *T. nitens* worker) escaped from the filming arenas during the small-nest experiment, so there are a total of 312 individual-level time series from the small-nest experiment instead of 320. These escaped workers were thus excluded from our analyses.

The four illustrative time series that appeared in the corners of our behavioral phenospace (main text Figure 2A) were generated by numerically simulating a FitzHugh-Nagumo model. The FitzHugh-Nagumo model was originally designed to describe the behavior of neural oscillations but is often used across disciplines as a generic formulation of an excitable system (10–12). Our use of this model here is not meant to provide any mechanistic insights into the process of synchronization in ants but merely illustrate the qualitative features of time series occupying different regions of the behavioral phenospace. The model consists of two differential equations; our noise-excited variant of this model is the same as the form presented by Pikovsky and Kurths (13).

$$\epsilon \frac{dx}{dt} = x - \frac{1}{3}x^3 - y \qquad (1)$$

$$\frac{dy}{dt} = x + a + D\xi(t) \tag{2}$$

In equations 1 and 2, the variables x and y represent the behavioral dynamics of the oscillator (membrane voltage and neuron recovery in the model's original context). The ξ term represents the application of Gaussian noise, and D is the amplitude of the added noise. The terms a and ϵ represent adjustable parameters of the systems. The example time series used in Figure 2A from the main text plot the y variable. We ran simulations of this model using the deSolve package in R.

The MATLAB function sort_nat was used to ensure that our image sequences were correctly sorted in chronological order for our image analyses. This function was downloaded from the MathWorks File Exchange: (https://www.mathworks.com/matlabcentral/fileexchange/10959-sort_nat-natural-order-sort).

SI figures

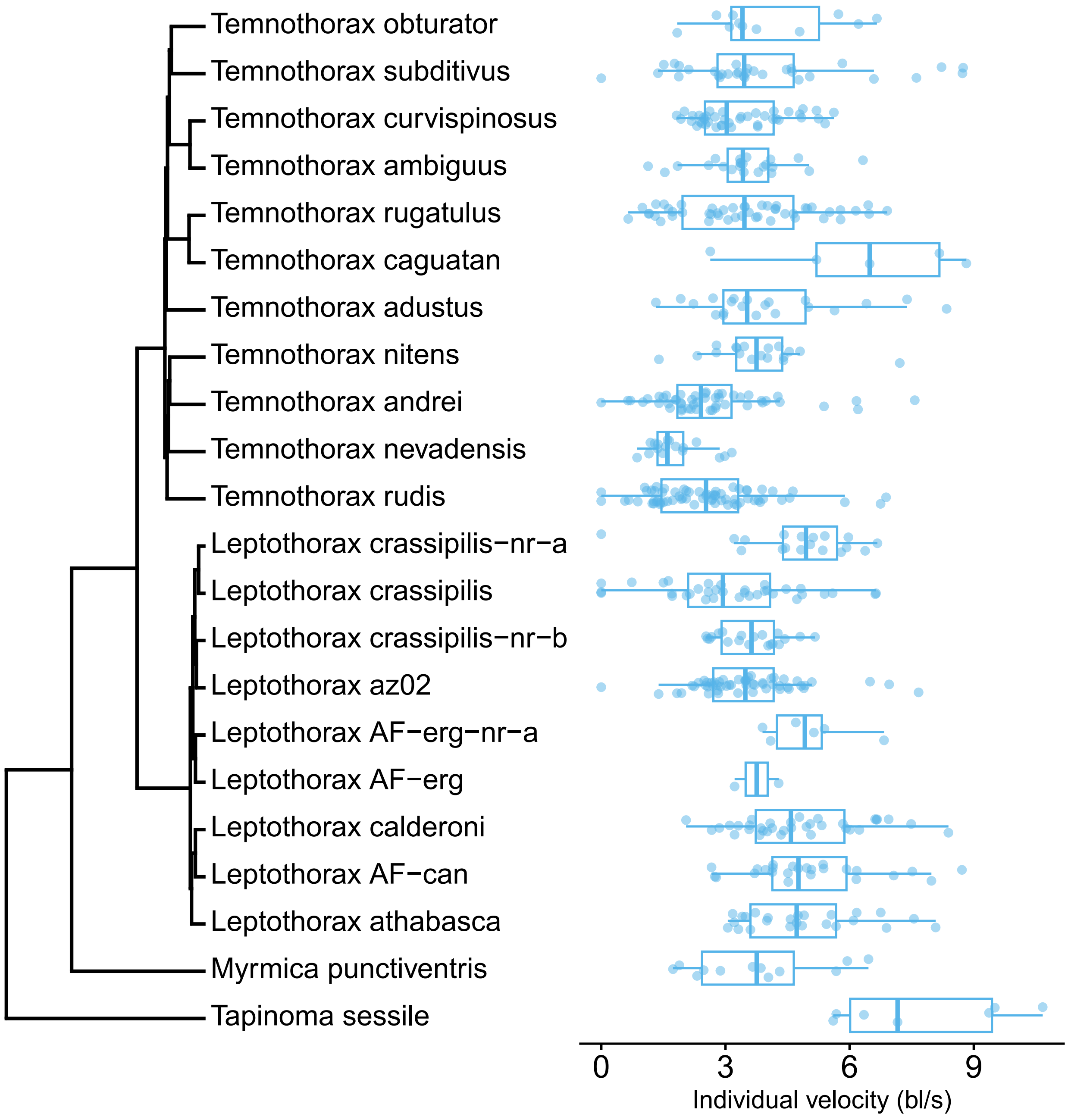

Figure S1. Species vary in the walking speed of isolated workers. The phylogeny of species used in this study, with boxplots showing the walking speed data from isolated individuals. Each data point represents the walking speed of a unique individual during her respective open field assay.

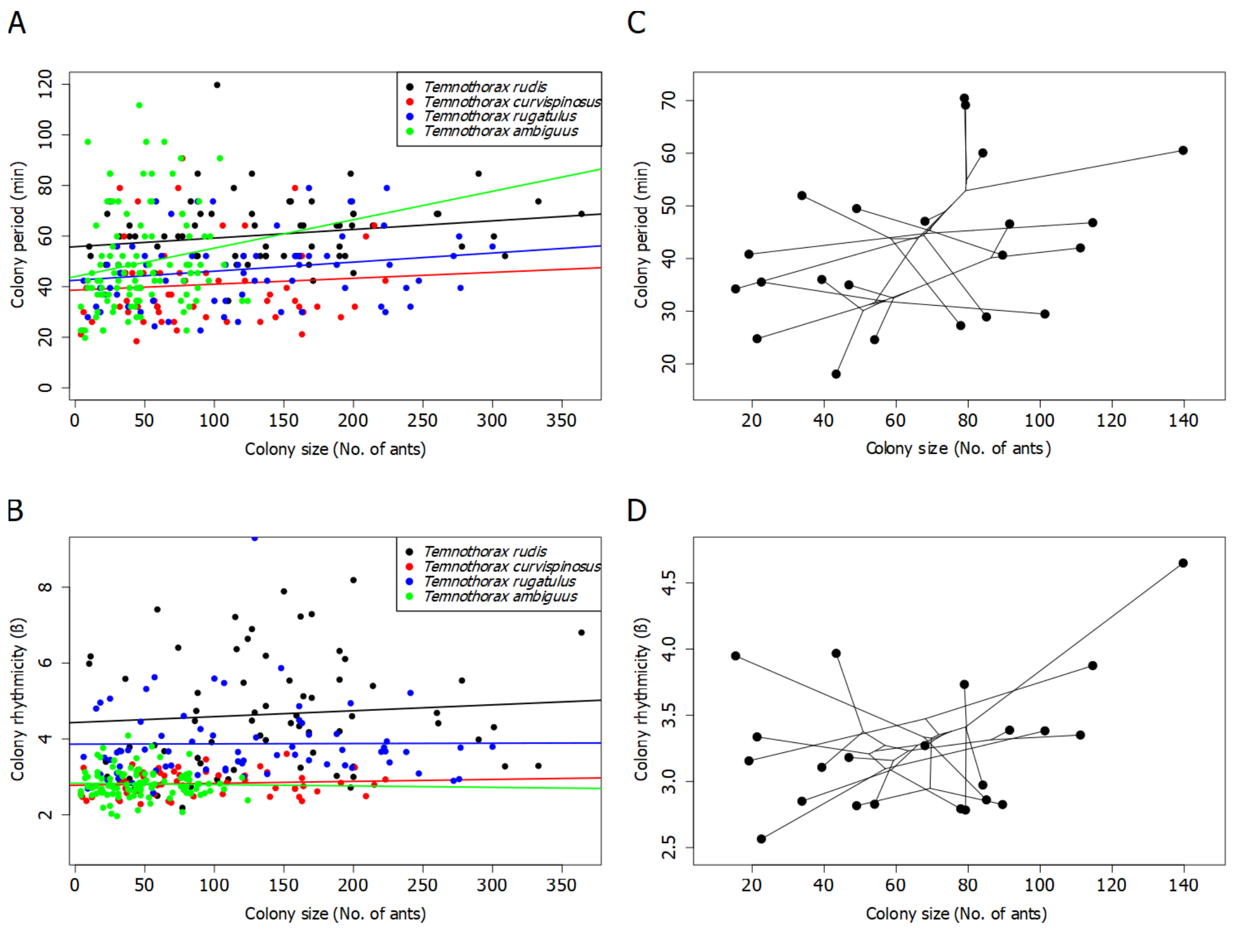

Figure S2. Colony size does not predict colony-level period or colony-level rhythmicity in our study. (a-b) Scatter plots of the dominant period or rhythmicity of colonies' collective activity vs. the colony size (i.e., approximate number of adult ants inside the nest at the time of recording). Data points are colored according to species. Each data point represents a different time series. Because most colonies were recorded twice, multiple datapoints thus correspond to the same colony measured on different days. The solid lines depict the LME regression for each species. None of the correlations were significant (p-value > 0.1 in all cases; see Dataset S8). (c-d) Phylomorphospace plots of the dominant period or rhythmicity of species' collective activity vs. the colony size. Each data point represents a different species. The values of these datapoints were the coefficient values obtained from fitting LME models with colony-level period or collective rhythmicity as the response variable and colony size as the fixed effect. Colony ID was used as a random effect in both models. Colony size was not correlated with either activity rhythm trait: colony-level period vs. colony size (PGLS: t-statistic = 1.23, p-value = 0.23) nor colony-level rhythmicity vs. colony size (PGLS: t-statistic = 1.90, p-value = 0.07).

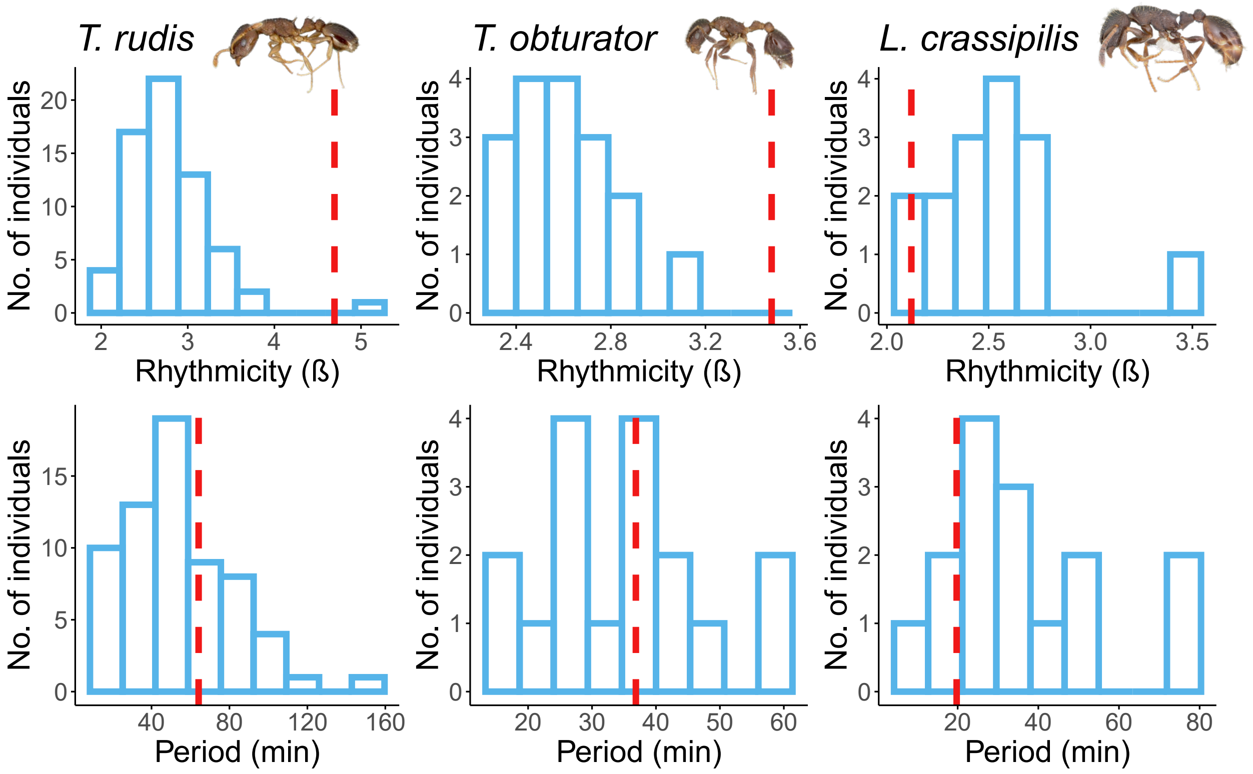

Figure S3. The colony-level rhythmicity of a given colony can significantly exceed the average individual-level rhythmicity of workers from that same colony. Histograms of individual-level trait distributions of the constituent ants from the three colonies used in the disassembly experiment. Each column corresponds to one of the three species (*T. rudis*, *T. obturator*, and *L. crassipilis*). The red dotted line in each panel represents the colony-level value for each trait. Specimen images are to scale and were extracted from images from www.antweb.org (casent0005689, casent0104756, and casent0104820).

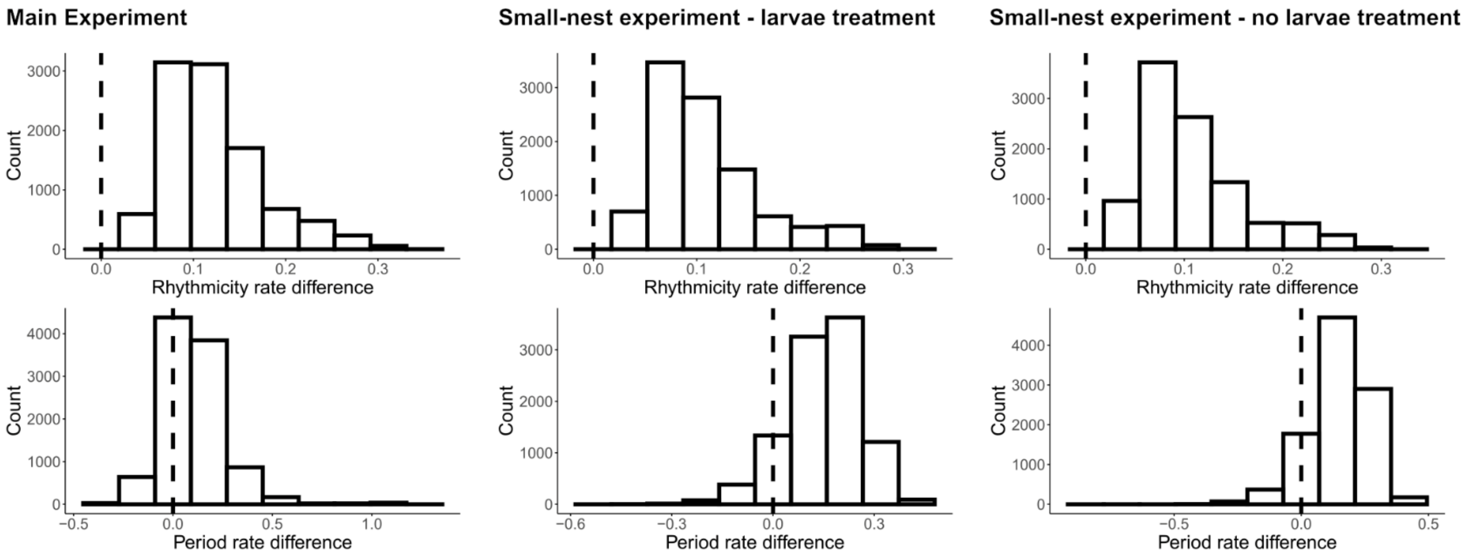

Figure S4. The difference in the estimated rates of evolution for colony-level vs. individual-level rhythmicity are significantly higher than the null expectation. Histograms of the rate differences (e.g., $\sigma^2_{Colony-level\ rhythmicity} - \sigma^2_{Individual-level\ rhythmicity}$) estimated using our bootstrap analyses for the main experiment, and the two treatments in the small-nest experiment. There are 10,000 rate difference estimates in each distribution. The dotted line in each panel represents the location of the null expectation of a rate difference of zero.

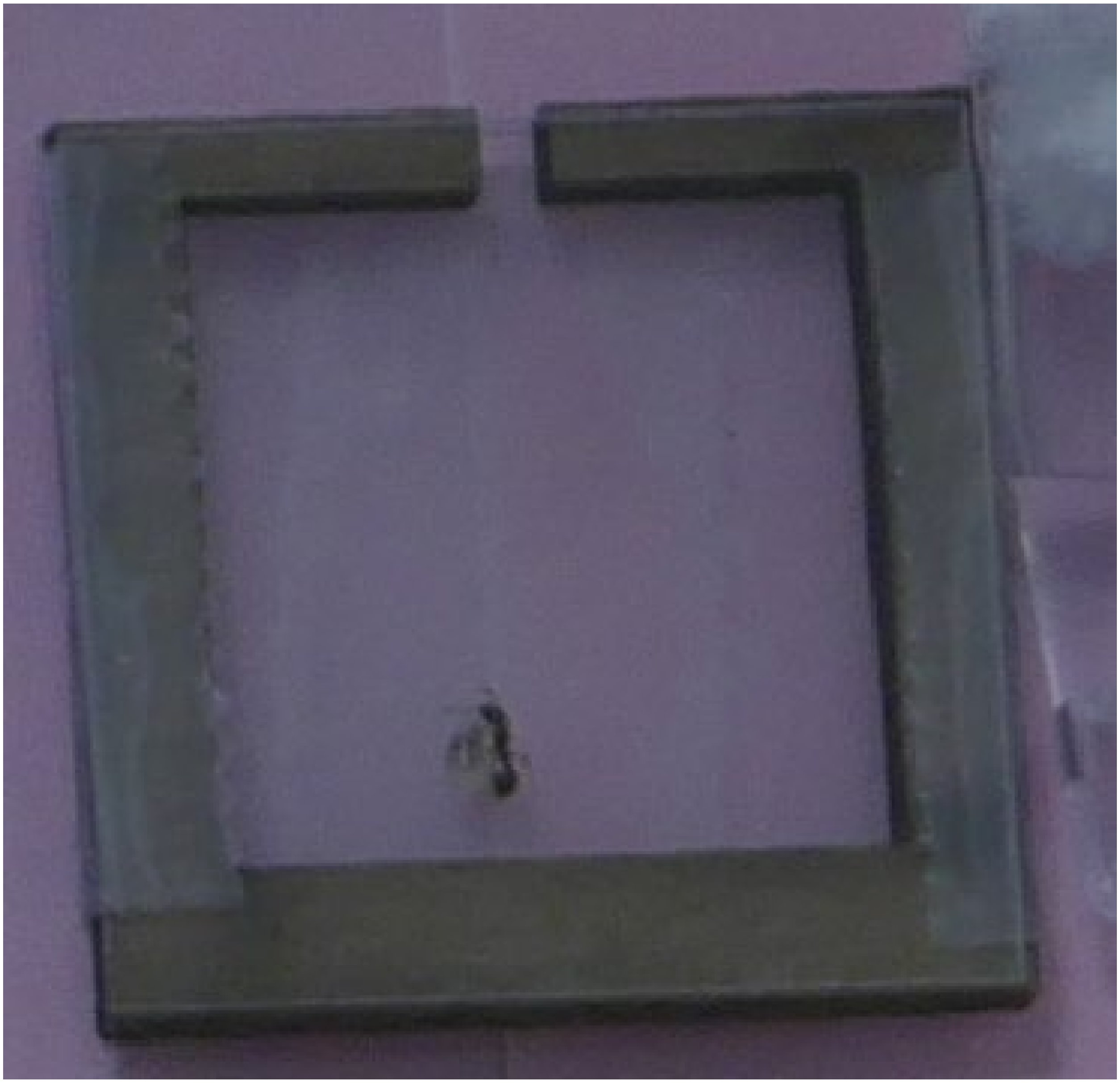

Figure S5. Isolated worker ants inhabited the artificial nests. A cropped frame from one of our recordings of an individual in our small-nest experiment who was isolated in an artificial nest with a larva. The isolated *Temnothorax rudis* worker in this frame is resting inside the nest next to the larva (to the left of the worker). The isolated workers in our experiments still alternated between states of rest and activity on ultradian timescales, and workers continued to exhibit normal behaviors post-isolation. For example, workers that were isolated with a larva typically rested inside the nest near the larvae (as can be seen in this image) and groomed the larva during their activity bouts.

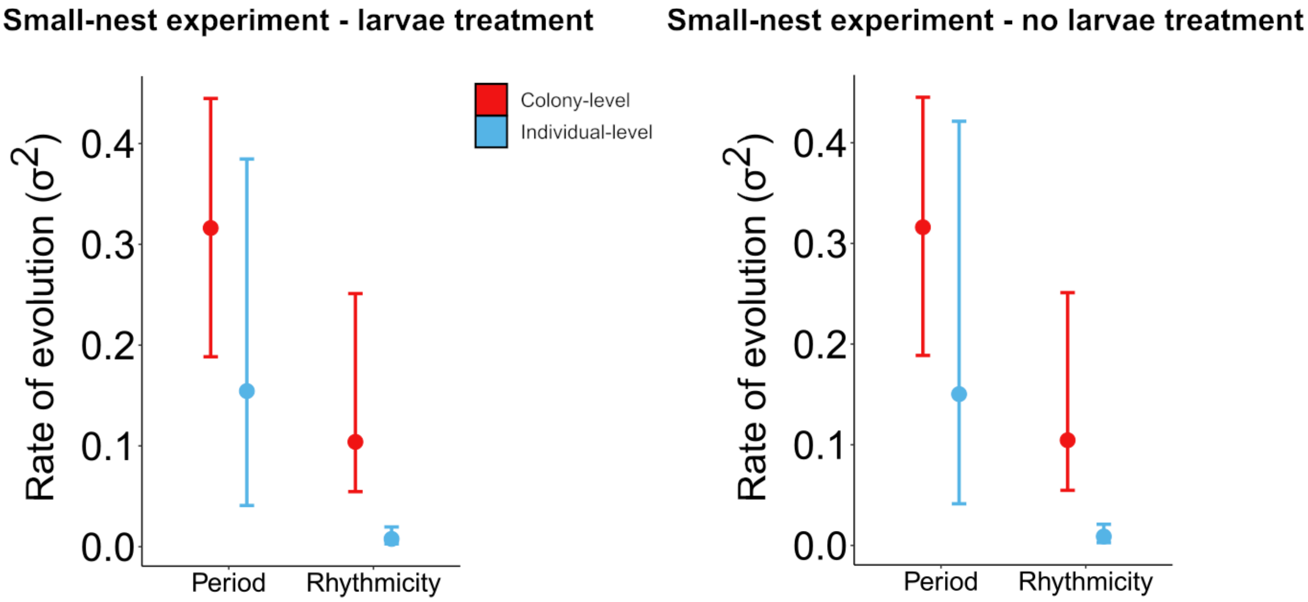

Figure S6. The results of our rate of evolution analysis from our small-nest experiment were consistent with the rate analysis from our main study: the rate of evolution (σ^2) for colony-level rhythmicity was significantly higher than the rate of evolution for individual-level rhythmicity. No significant difference was found between the rates of colony-level period vs. individual-level period. The set of rate estimates for each trait was obtained using a bootstrapping approach (see main text). Dots represent rate medians and error bars depict the 95% confidence intervals.

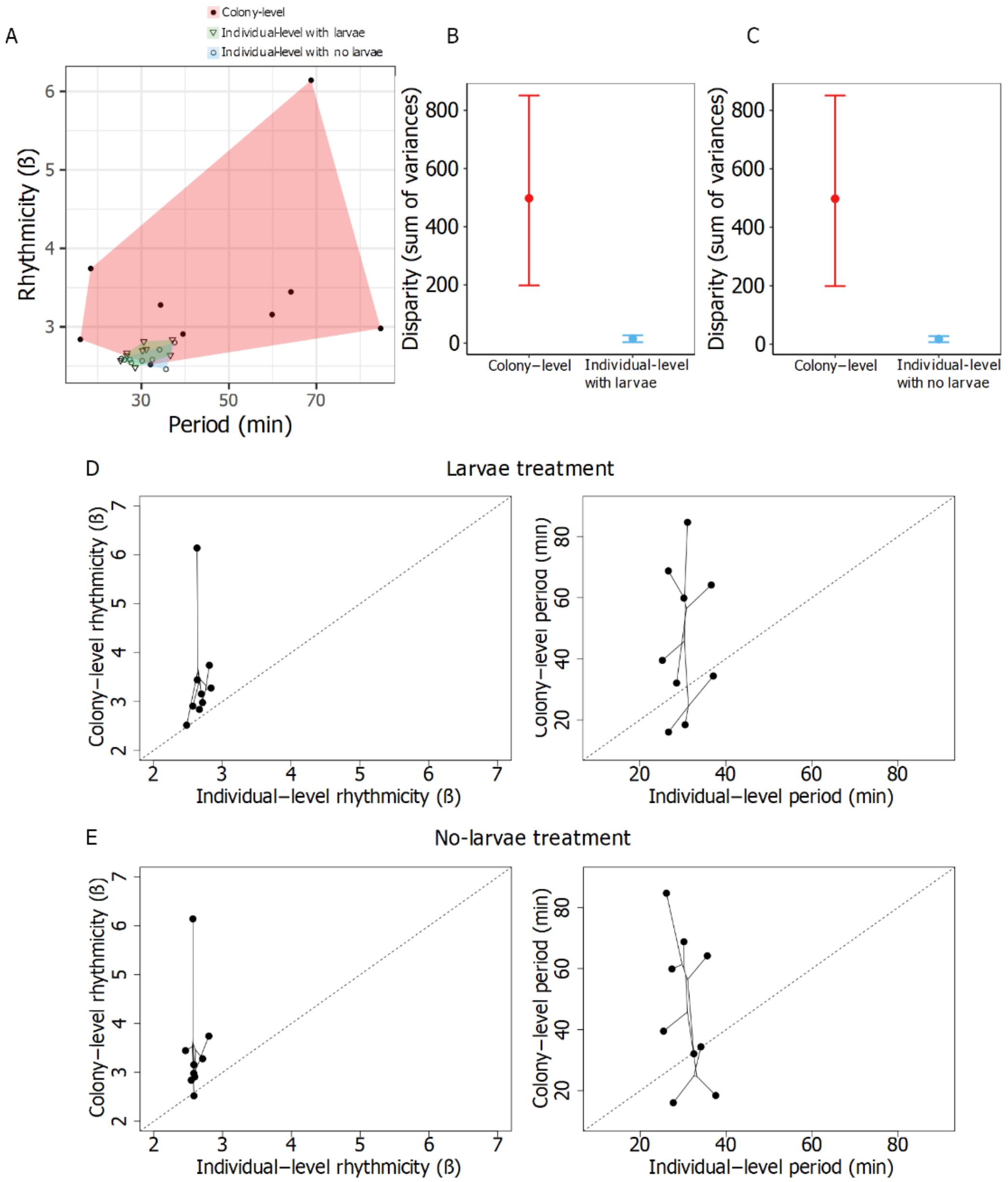

Figure S7. The results of our phenotypic disparity and phylomorphospace analyses from our small-nest experiment were consistent with the same analyses from our main study: the phenotypic disparity of colony-level rhythm traits was significantly larger than the phenotypic disparity of individual-level traits, and there was no correlation between a species' individual-level traits and their corresponding colony-level traits. (a) Behavioral phenospace of ant ultradian rhythms across the 9 different species used in our small-nest experiment. Each datapoint represents a different species. Solid points falling within the pink polygon correspond to the colony-level rhythms of the 9 colonies recorded specifically for the small-nest experiment, the open circles within the blue polygon correspond to these species' individual-level rhythms in the no-larvae treatment, and the open triangles within the green polygon correspond to these species' individual-level rhythms in the larvae treatment. (b-c)

comparison of the phenotypic disparity (measured as the sum of variance) between species' colony-level and individual-level behavioral phenospaces from the two treatments in the small-nest experiment. The sum of variance metrics were calculated by bootstrapping the phenospace data. Dots represent medians and error bars depict the 95% confidence intervals. (d) Behavioral phylomorphospaces for individual-level vs. colony-level traits for the larvae treatment and (e) individual-level vs. colony-level traits for the no-larvae treatment. The dotted lines show what a 1:1 relationship between the variables would be.

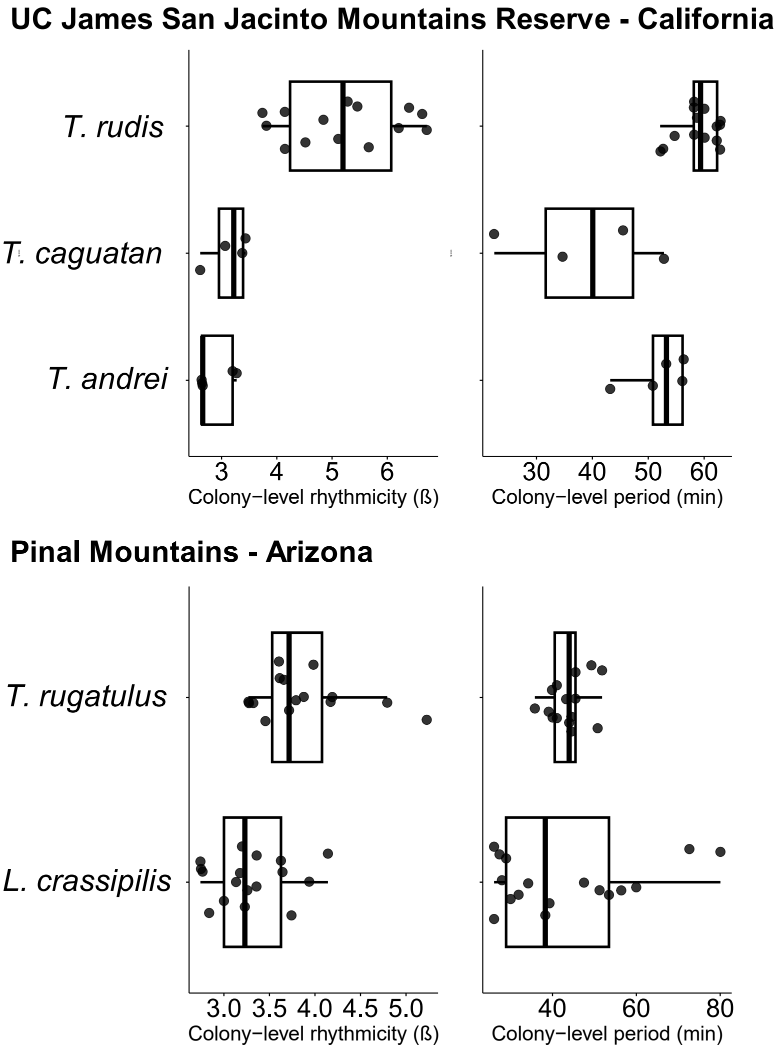

Figure S8. Colonies from different species living at the same site can exhibit significantly different colony-level rhythm traits. Boxplots depicting the colony-level period and colony-level rhythmicity traits for different species collected at two different sites. Each datapoint in a given box represents the trait mean across observations of a unique colony. Colonies of *Temnothorax rudis*, *T. caguatan*, and *T. andrei* from the same location within the San Jacinto Mountains of California differ significantly in both their colony-level rhythmicity (LME model: numDF = 2, denDF = 20, F-value = 19.9572, p-value < 0.0001) as well as their colony-level period traits (LME model: numDF = 2, denDF = 20, F-value = 8.8602, p-value = 0.0018). In the Pinal Mountains of Arizona, *Temnothorax rugatulus* and *Leptothorax crassipilis* both occur at the same site and nest in the same type of granite rock crevices, yet they differ significantly in their colony-level rhythmicity trait (LME model: numDF = 1, denDF = 30, F-value = 10.4943, p-value = 0.0029).

Legends for Movie S1 and Legends for Datasets S1 to S14

Movie S1.
A 14.5 hour recording of one of the colonies used in the study (colony ID: JRCT4, Species: *Temnothorax rudis*) along with a concurrent animation of the collective activity time series associated with this particular recording. The recording has been sped up by approximately 153,429% to help easily visualize the cyclic activity of the colony.

Dataset S1.
The ultradian behavioral metrics from all colony-level time series used in the main experiment. Each row corresponds to a different colony-level time series. Collection locality details and the date of collection for each colony are also provided in this table. The approximate number of ants in each nest at the time of each recording is also listed on each row. The columns with "trim" in their heading denote behavioral metrics calculated with the 8-hour colony-level time series.

Dataset S2.
The ultradian behavioral metrics from all individual-level time series used in the main experiment.

Dataset S3.
The mean moving velocity metrics from all individuals used in the open field assay.

Dataset S4.
The statistical results of our LME models assessing interspecific variation in the five behavioral metrics from our main experiment.

Dataset S5.
The list of voucher specimens that provided genetic material for this study.

Dataset S6.
A table of sequencing information for each taxon in the phylogeny. Rows listed as being from "this study" in the "reference" column indicate that new sequence data from these samples was obtained for the present study. These individual ants were not necessarily used for the behavioral experiments.

Dataset S7.
A table containing calibration details for our phylogeny.

Dataset S8.
The statistical results of our supplementary colony size analysis.

Dataset S9.
The ultradian behavioral metrics from all colony-level time series from the disassembly experiment. Each row corresponds to a different colony-level time series.

Dataset S10.
The ultradian behavioral metrics from all individual-level time series from the disassembly experiment. Each row corresponds to a different individual-level time series.

Dataset S11.
The ultradian behavioral metrics from all colony-level time series used in the small-nest experiment. Each row corresponds to a different colony-level time series.

Dataset S12.
The ultradian behavioral metrics from all individual-level time series used in the small-nest experiment. Each row corresponds to a different individual-level time series.

Dataset S13.
A summary table that lists the number of unique colonies/individuals of each species as well as the individual-level and colony-level activity trait estimates for each species used in the main experiment and the small-nest experiment. The activity trait estimates were calculated for the full length of each time-series using the procedures described in the main text and SI methods.

Dataset S14.
The code for our statistical analyses written in the R language and presented as the html output of an R markdown file.

SI references

1. G. N. Doering, *et al.*, Sources of intraspecific variation in the collective tempo and synchrony of ant societies. *Behav Ecol* **30**, 1682–1690 (2019).

2. B. J. Cole, L. Hoeg, The influence of brood type on activity cycles in *Leptothorax allardycei* (Hymenoptera: Faruicidae). *J Insect Behav* **9**, 539–547 (1996).

3. N. Pinter-Wollman, Personality in social insects: How does worker personality determine colony personality? *Curr Zool* **58**, 580–588 (2012).

4. G. N. Doering, *et al.*, Noise resistant synchronization and collective rhythm switching in a model of animal group locomotion. *Royal Society Open Science* **9**, 211908 (2022).

5. B. J. Cole, Short-Term Activity Cycles in Ants: Generation of Periodicity by Worker Interaction. *The American Naturalist* **137**, 244–259 (1991).

6. G. N. Doering, C. L. Lee, K. Dalnoki-Veress, Synchronized locomotion can improve spatial accessibility inside ant colonies. *Proceedings of the Royal Society B: Biological Sciences* **290**, 20231805 (2023).

7. G. Farnebäck, Two-Frame Motion Estimation Based on Polynomial Expansion in *Image Analysis*, Lecture Notes in Computer Science., J. Bigun, T. Gustavsson, Eds. (Springer, 2003), pp. 363–370.

8. E. O. Wilson, The Population Consequences of Polygyny in the Ant *Leptothorax curvispinosus* Mayr. *Annals of the Entomological Society of America* **67**, 781–786 (1974).

9. A. Buschinger, *Verbreitung und Auswirkungen von Mono-und Polygynie bei Arten der Gattung* Leptothorax *Mayr (Hymenoptera, Formicidae)* (Institut für Angewandte Zoologie, Universität Würzburg, 1967).

10. J. Keener, J. Sneyd, Eds., “Excitability” in *Mathematical Physiology*, Interdisciplinary Applied Mathematics., (Springer, 1998), pp. 116–159.

11. R. FitzHugh, Mathematical models of threshold phenomena in the nerve membrane. *Bulletin of Mathematical Biophysics* **17**, 257–278 (1955).

12. J. Nagumo, S. Arimoto, S. Yoshizawa, An Active Pulse Transmission Line Simulating Nerve Axon. *Proceedings of the IRE* **50**, 2061–2070 (1962).

13. A. S. Pikovsky, J. Kurths, Coherence Resonance in a Noise-Driven Excitable System. *Phys. Rev. Lett.* **78**, 775–778 (1997).
