# Supplementary material for: Emergent collective behavior evolves more rapidly than individual behavior among acorn ant species
Source: Proc Natl Acad Sci U S A. 2024 Nov 22;121(48):e2420078121. doi: 10.1073/pnas.2420078121 (PMC11621464; doi:10.1073/pnas.2420078121)
Supplement: Supplementary file 15 — Dataset S14 (PDF) [file pnas.2420078121.sd14.pdf]

Comparative analyses of acorn ant ultradian rhythms

Doering, Prebus, Suresh, Greer, Bowden, Linksvayer

9/29/2024

- Import data
- Interspecific differences
- Phylogenetic regression
- Behavioral phenospace analyses
- Rate of evolution
- Disassembly analyses
- Supplementary colony size analysis
- Small-nest analyses
- Locality analysis


```
set.seed(9) # set the rng for reproducibility
setwd("C:/Users/naviddio/OneDrive/CA_PCM/compile/data")

library(nlme)
library(stringr)
library(ape)
library(geiger)
library(phytools)
library(dplyr)
library(picante)
library(adephylo)
library(ade4)
library(phylobase)
library(dispRity)
library(sensiPhy)
library(gdata)
library(plotrix)
```

Import data

```
CR <- read.csv("Table_S1_collective_activity.csv", header=T)
IR <- read.csv("Table_S2_individual_activity.csv", header=T)
EX <- read.csv("Table_S3_individual_velocity.csv", header=T)
CR_deconstruct <- read.csv("Table_S9_collective_activity_disassembly.csv", header=T)
IR_deconstruct <- read.csv("Table_S10_individual_activity_disassembly.csv", header=T)
```

Interspecific differences

Here, we use LME models to assess interspecific differences in the five behavioral traits considered for this study. Colony ID (or Colony of origin for individual ants) is set as a random effect in all of the models. The model coefficient estimates

are used to obtain tip-values for each of the species in our phylogenetic analyses.

```
m1 <- lme(Dominant_Period ~ Species, random=~1|Colony, data=CR) # model for collective-level p
eriod
anova(m1)
```

```
##             numDF denDF  F-value p-value
## (Intercept)     1   292 3660.253  <.0001
## Species        21   242    9.841  <.0001
```

```
period.CR=data.frame(summary(m1)$tTable)
period.CR$Value[2:length(period.CR$Value)]=period.CR$Value[2:length(period.CR$Value)]+period.C
R$Value[1]
row.names(period.CR)=sort(unique(CR$Species))

m1 <- lme(cf_wavelet ~ Species, random=~1|Colony, data=CR) # model for collective-level rhythm
icity
anova(m1)
```

```
##             numDF denDF  F-value p-value
## (Intercept)     1   292 8592.448  <.0001
## Species        21   242   14.335  <.0001
```

```
wavelet.CR=data.frame(summary(m1)$tTable)
wavelet.CR$Value[2:length(wavelet.CR$Value)]=wavelet.CR$Value[2:length(wavelet.CR$Value)]+wave
let.CR$Value[1]
row.names(wavelet.CR)=sort(unique(CR$Species))

m1 <- lme(Dominant_Period ~ Species, random=~1|Colony, data=IR) # model for individual-level p
eriod
anova(m1)
```

```
##             numDF denDF   F-value p-value
## (Intercept)     1   455 1897.0894  <.0001
## Species        21    79    3.3596  <.0001
```

```
period.IR=data.frame(summary(m1)$tTable)
period.IR$Value[2:length(period.IR$Value)]=period.IR$Value[2:length(period.IR$Value)]+period.I
R$Value[1]
row.names(period.IR)=sort(unique(CR$Species))

m1 <- lme(cf_wavelet ~ Species, random=~1|Colony, data=IR) # model for individual-level rhythm
icity
anova(m1)
```

```
##             numDF denDF  F-value p-value
## (Intercept)     1   455 36667.57  <.0001
## Species        21    79     1.46  0.1191
```

```
wavelet.IR=data.frame(summary(m1)$tTable)
wavelet.IR$Value[2:length(wavelet.IR$Value)]=wavelet.IR$Value[2:length(wavelet.IR$Value)]+wave
let.IR$Value[1]
row.names(wavelet.IR)=sort(unique(CR$Species))

m1 <- lme(Mean_moving_velocity ~ Species, random=~1|Colony, data=EX) # model for individual-le
vel velocity
anova(m1)
```

```
##             numDF denDF   F-value p-value
## (Intercept)     1   473 1236.4950  <.0001
## Species        21    79    5.1888  <.0001
```

```
velocity.EX=data.frame(summary(m1)$tTable)
velocity.EX$Value[2:length(velocity.EX$Value)]=velocity.EX$Value[2:length(velocity.EX$Value)]+
velocity.EX$Value[1]
row.names(velocity.EX)=sort(unique(CR$Species))
```

Phylogenetic regression

This code section runs PGLS and a sensitivity analysis to test for correlations between analogous pairs of individual-level and collective-level traits.

```
t<- read.nexus("doering_25t_divergence_names.tre") # read tree file

nc <- name.check(t, wavelet.CR)

pruned <- drop.tip(t, nc$tree_not_data)
pruned<-force.ultrametric(pruned) # The numerical precision of the branch lengths in our phylo
geny result in it not being recognized as an ultrametric tree by R. We therefore apply the for
ce.ultrametric function to ensure that our tree is treated as ultrametric.

CR$Dominant_Period<-CR$Dominant_Period_trim # ensure that the trait values for the shorter col
lective-level time series are used to match the length of the individual-level time series
CR$cf_wavelet<-CR$cf_wavelet_trim

m1 <- lme(Dominant_Period ~ Species, random=~1|Colony, data=CR)

period.CR=data.frame(summary(m1)$tTable)
period.CR$Value[2:length(period.CR$Value)]=period.CR$Value[2:length(period.CR$Value)]+period.C
R$Value[1]
row.names(period.CR)=sort(unique(CR$Species))

m1 <- lme(cf_wavelet ~ Species, random=~1|Colony, data=CR)

wavelet.CR=data.frame(summary(m1)$tTable)
wavelet.CR$Value[2:length(wavelet.CR$Value)]=wavelet.CR$Value[2:length(wavelet.CR$Value)]+wave
let.CR$Value[1]
row.names(wavelet.CR)=sort(unique(CR$Species))
```

```
wavelet.CR.value=setNames(wavelet.CR$Value,row.names(wavelet.CR)) #save tip-values for each sp
ecies' traits from LME models
wavelet.IR.value=setNames(wavelet.IR$Value,row.names(wavelet.IR))
period.CR.value=setNames(period.CR$Value,row.names(period.CR))
period.IR.value=setNames(period.IR$Value,row.names(period.IR))
velocity.EX.value=setNames(velocity.EX$Value,row.names(velocity.EX))

wavelet.CR.SE=setNames(wavelet.CR$Std.Error,row.names(wavelet.CR)) #save standard error of eac
h species' traits from LME models
wavelet.IR.SE=setNames(wavelet.IR$Std.Error,row.names(wavelet.IR))
period.CR.SE=setNames(period.CR$Std.Error,row.names(period.CR))
period.IR.SE=setNames(period.IR$Std.Error,row.names(period.IR))
velocity.EX.SE=setNames(velocity.EX$Std.Error,row.names(velocity.EX))

X_traits<-data.frame(wavelet.CR.value,period.CR.value,wavelet.IR.value,period.IR.value,velocit
y.EX.value,wavelet.CR.SE,wavelet.IR.SE,period.CR.SE, period.IR.SE, velocity.EX.SE)
row.names(X_traits)<-row.names(period.CR)
X_traits$Species<-rownames(X_traits)

CR3<-data.frame(period.CR.value,wavelet.CR.value)
colnames(CR3)<-c("Dominant_Period","cf_wavelet")
CR3$Species<-rownames(CR3)
IR3<-data.frame(period.IR.value,wavelet.IR.value)
colnames(IR3)<-c("Dominant_Period","cf_wavelet")
IR3$Species<-rownames(IR3)

combined<-combine(CR3,IR3)
```

```
set.seed(9)
intra <- intra_phylm(period.CR.value ~ period.IR.value,
                     phy = pruned, data = X_traits, Vx = "period.IR.SE", Vy = "period.CR.SE",
n.intra = 10000)
```

```
summary(intra)
```

```
##                   mean CI_low CI_high
## intercept       26.829 26.668  26.991
## se.intercept    10.964 10.917  11.011
## pval.intercept   0.056  0.054   0.058
## estimate         0.323  0.317   0.330
## se.estimate      0.347  0.345   0.348
## pval.estimate    0.379  0.373   0.385
```

```
pglsModel <- gls(period.CR.value  ~ period.IR.value , correlation = corBrownian(phy = pruned,
form = ~Species), data = X_traits, method = "ML")
summary(pglsModel)
```

```
## Generalized least squares fit by maximum likelihood
##   Model: period.CR.value ~ period.IR.value
##   Data: X_traits
##        AIC      BIC    logLik
```

```
##   173.3868 176.6599 -83.69339
##
## Correlation Structure: corBrownian
##  Formula: ~Species
##  Parameter estimate(s):
## numeric(0)
##
## Coefficients:
##                     Value Std.Error   t-value p-value
## (Intercept)     31.329847 18.824755 1.6642897  0.1116
## period.IR.value  0.114349  0.388266 0.2945128  0.7714
##
##  Correlation:
##                 (Intr)
## period.IR.value -0.487
##
## Standardized residuals:
##         Min          Q1         Med          Q3         Max
## -0.60619898 -0.31956665  0.03786284  0.34916317  0.88178380
##
## Residual standard error: 25.67424
## Degrees of freedom: 22 total; 20 residual
```

```
set.seed(9)
intra <- intra_phylm(wavelet.CR.value ~ wavelet.IR.value,
                     phy = pruned, data = X_traits, Vx = "wavelet.IR.SE", Vy = "wavelet.CR.SE"
, n.intra = 10000)
```

```
summary(intra)
```

```
##                   mean CI_low CI_high
## intercept        3.294  3.250   3.339
## se.intercept     2.193  2.184   2.203
## pval.intercept   0.250  0.244   0.255
## estimate        -0.016 -0.032   0.001
## se.estimate      0.810  0.807   0.814
## pval.estimate    0.498  0.492   0.504
```

```
pglsModel <- gls(wavelet.CR.value  ~ wavelet.IR.value , correlation = corBrownian(phy = pruned
, form = ~Species), data = X_traits, method = "ML")
summary(pglsModel)
```

```
## Generalized least squares fit by maximum likelihood
##   Model: wavelet.CR.value ~ wavelet.IR.value
##   Data: X_traits
##        AIC      BIC    logLik
##   43.46016 46.73329 -18.73008
##
## Correlation Structure: corBrownian
##  Formula: ~Species
```

```
##  Parameter estimate(s):
## numeric(0)
##
## Coefficients:
##                        Value Std.Error    t-value p-value
## (Intercept)        3.1069955  3.262957  0.9522023  0.3524
## wavelet.IR.value -0.0151887  1.142013 -0.0133000  0.9895
##
##  Correlation:
##                  (Intr)
## wavelet.IR.value -0.965
##
## Standardized residuals:
##         Min          Q1         Med          Q3         Max
## -0.28557626 -0.06077338  0.05801386  0.27588814  0.91591520
##
## Residual standard error: 1.339921
## Degrees of freedom: 22 total; 20 residual
```

Behavioral phenospace analyses

This code section calculates the phenotypic disparity of collective-level behavior and individual-level behavior. We perform a bootstrap analysis to compare the differences between the estimated disparity values of the two levels against the null expectation of a difference of zero.

```
CR3<-data.frame(period.CR.value,wavelet.CR.value)
colnames(CR3)<-c("Dominant_Period","cf_wavelet")
CR3$Species<-rownames(CR3)
IR3<-data.frame(period.IR.value,wavelet.IR.value)
colnames(IR3)<-c("Dominant_Period","cf_wavelet")
IR3$Species<-rownames(IR3)

combined<-combine(CR3,IR3)

X<-subset(combined, select = -c(Species, source))

tier<-custom.subsets(X, list(Colony = c(1:22), Individual = c(23:44)))
set.seed(9)
tier_bootstrap<-boot.matrix(tier,bootstrap=10000) # acquire bootstrap dataset using the dispRi
ty package

tier_disparity<-dispRity(tier_bootstrap,metric=c(sum,variances)) # compute disparity metric fo
r collective-level and individual-level behavior in the bootstrapped dataset

ind_boot_disparity<-as.data.frame(tier_disparity$disparity$Individual[2])
ind_boot_disparity<-t(ind_boot_disparity)
colony_boot_disparity<-as.data.frame(tier_disparity$disparity$Colony[2])
colony_boot_disparity<-t(colony_boot_disparity)
ind_boot_disparity<-as.data.frame(ind_boot_disparity)
colony_boot_disparity<-as.data.frame(colony_boot_disparity)

Individual<-ind_boot_disparity$V1
```

```
Colony<-colony_boot_disparity$V1
boot_disparity<-combine(Colony,Individual)
boot_disparity$source = paste0(boot_disparity$source,"-level")

colnames(boot_disparity)[1]<-c("Sum_of_variances")

disparity_diff<-boot_disparity$Sum_of_variances[1:10000]-boot_disparity$Sum_of_variances[10001
:20000] # compute differences in disparity from the bootstrapped data

sum(0>disparity_diff) / length(disparity_diff) # compare observed differences against the null
 expectation of a difference of 0.
```

```
## [1] 1e-04
```

Rate of evolution

This code section performs our evolutionary rate analyses. We perform bootstrap analyses that compare the differences between the estimated rates of evolution for analogous pairs of collective-level and individual-level traits against the null expectation of a rate difference of zero.The for loop at the beginning of this code section can be un-commented to acquire the 10,000 rate estimate samples for each trait. However, it is also possible to load the previously completed bootstrap rate data to avoid re-running the computationally time-consuming bootstrapping procedure.

```
df2<-NULL
set.seed(9)
# for (i in 1:10){
#
#   CR<-as.data.frame(CR)
#   IR<-as.data.frame(IR)
#   EX<-as.data.frame(EX)
#   CR.sample<-sample_n(CR, nrow(CR), replace = TRUE)
#   CR.sample<-as.data.frame(CR.sample)
#   IR.sample<-sample_n(IR, nrow(IR), replace = TRUE)
#   IR.sample<-as.data.frame(IR.sample)
#   EX.sample<-sample_n(EX, nrow(EX), replace = TRUE)
#   EX.sample<-as.data.frame(EX.sample)
#   m.period.CR <- lme(Dominant_Period ~ Species, random=~1|Colony, data=CR.sample)
#   m.wavelet.CR <- lme(cf_wavelet ~ Species, random=~1|Colony, data=CR.sample)
#   m.period.IR <- lme(Dominant_Period ~ Species, random=~1|Colony, data=IR.sample)
#   m.wavelet.IR <- lme(cf_wavelet ~ Species, random=~1|Colony, data=IR.sample)
#   m.velocity.EX <- lme(Mean_moving_velocity  ~ Species, random=~1|Colony, data=EX.sample)
#
#   period.CR=data.frame(summary(m.period.CR)$tTable)
#   period.CR$Value[2:length(period.CR$Value)]=period.CR$Value[2:length(period.CR$Value)]+peri
od.CR$Value[1]
#   row.names(period.CR)=sort(unique(CR.sample$Species))
#
#   wavelet.CR=data.frame(summary(m.wavelet.CR)$tTable)
#   wavelet.CR$Value[2:length(wavelet.CR$Value)]=wavelet.CR$Value[2:length(wavelet.CR$Value)]+
wavelet.CR$Value[1]
#   row.names(wavelet.CR)=sort(unique(CR.sample$Species))
#
```

```
#   period.IR=data.frame(summary(m.period.IR)$tTable)
#   period.IR$Value[2:length(period.IR$Value)]=period.IR$Value[2:length(period.IR$Value)]+peri
od.IR$Value[1]
#   row.names(period.IR)=sort(unique(IR.sample$Species))
#
#   wavelet.IR=data.frame(summary(m.wavelet.IR)$tTable)
#   wavelet.IR$Value[2:length(wavelet.IR$Value)]=wavelet.IR$Value[2:length(wavelet.IR$Value)]+
wavelet.IR$Value[1]
#   row.names(wavelet.IR)=sort(unique(IR.sample$Species))
#
#   velocity.EX=data.frame(summary(m.velocity.EX)$tTable)
#   velocity.EX$Value[2:length(velocity.EX$Value)]=velocity.EX$Value[2:length(velocity.EX$Valu
e)]+velocity.EX$Value[1]
#   row.names(velocity.EX)=sort(unique(EX.sample$Species))
#
#   wavelet.CR.value=setNames(wavelet.CR$Value,row.names(wavelet.CR))/mean(wavelet.CR$Value) #
 standardize the behavioral metrics
#   wavelet.IR.value=setNames(wavelet.IR$Value,row.names(wavelet.IR))/mean(wavelet.IR$Value)
#   period.CR.value=setNames(period.CR$Value,row.names(period.CR))/mean(period.CR$Value)
#   period.IR.value=setNames(period.IR$Value,row.names(period.IR))/mean(period.IR$Value)
#   velocity.EX.value=setNames(velocity.EX$Value,row.names(velocity.EX))/mean(velocity.EX$Valu
e)
#
#
#   nc_p<-as.data.frame(unique(CR.sample$Species))
#   row.names(nc_p)<-nc_p$`unique(CR.sample$Species)`
#   nc <- name.check(t, nc_p)
#   pruned.CR <- drop.tip(t, nc$tree_not_data)
#   pruned.CR<-force.ultrametric(pruned.CR)
#
#
#   nc_p<-as.data.frame(unique(IR.sample$Species))
#   row.names(nc_p)<-nc_p$`unique(IR.sample$Species)`
#   nc <- name.check(t, nc_p)
#   pruned.IR <- drop.tip(t, nc$tree_not_data)
#   pruned.RR<-force.ultrametric(pruned.IR)
#
#
#   nc_p<-as.data.frame(unique(EX.sample$Species))
#   row.names(nc_p)<-nc_p$`unique(EX.sample$Species)`
#   nc <- name.check(t, nc_p)
#   pruned.EX <- drop.tip(t, nc$tree_not_data)
#   pruned.EX<-force.ultrametric(pruned.EX)
#
#
#   # fit BM models of evolution for each trait and extract the rate parameter from each model
#
#   fc.wavelet.CR=fitContinuous(pruned.CR,dat=wavelet.CR.value,model="BM")
#   fc.wavelet.CR$opt
#
#   fc.wavelet.IR=fitContinuous(pruned.IR,dat=wavelet.IR.value,model="BM")
#   fc.wavelet.IR$opt
#
#   fc.period.CR=fitContinuous(pruned.CR,dat=period.CR.value,model="BM")
```

```
#   fc.period.CR$opt
#
#   fc.period.IR=fitContinuous(pruned.IR,dat=period.IR.value,model="BM")
#   fc.period.IR$opt
#
#   fc.velocity.EX=fitContinuous(pruned.EX,dat=velocity.EX.value,model="BM")
#   fc.velocity.EX$opt
#
#   df3<-data.frame(fc.wavelet.CR$opt[1],fc.wavelet.IR$opt[1],fc.period.CR$opt[1],fc.period.IR
$opt[1],0,fc.velocity.EX$opt[1])
#   colnames(df3)<-c("sigsq.wavelet.CR","sigsq.wavelet.IR", "sigsq.period.CR", "sigsq.period.I
R", "sigsq.velocity.placeholder","sigsq.velocity.EX")
#   df3<-t(df3)
#   df3<-data.frame(df3)
#   colnames(df3)<-c("sigsq")
#   df3$sigsq.tier<-rownames(df3)
#   df3$source<-c("Colony", "Individual","Colony", "Individual", "Colony", "Individual")
#   df3$trait<-c("Rhythmicity", "Rhythmicity","Period", "Period", "Velocity", "Velocity")
#
#   df2<-rbind(df2,df3)
#
# }

df2<-read.csv("rates_resample.csv", header = TRUE) # load previously completed bootstrap rate
data to avoid re-running the bootstrapping

x<-subset(df2, sigsq.tier == "sigsq.wavelet.CR")
x2<-subset(df2, sigsq.tier == "sigsq.wavelet.IR")
wavelet_r_diff<-x$sigsq-x2$sigsq # compute differences in the evolutionary rates of collective
-level and individual-level rhythmicity from the bootstrapped data
x<-subset(df2, sigsq.tier == "sigsq.period.CR")
x2<-subset(df2, sigsq.tier == "sigsq.period.IR")
period_r_diff<-x$sigsq-x2$sigsq  # compute differences in the evolutionary rates of collective
-level and individual-level period from the bootstrapped data

sum(0>period_r_diff) / length(period_r_diff) # compare observed rate differences against the n
ull expectation of a difference of 0.
```

```
## [1] 0.2288
```

```
sum(0>wavelet_r_diff) / length(wavelet_r_diff)
```

```
## [1] 0
```

Disassembly analyses

This code section loads the collective-level and individual-level time series from our disassembly experiment and compares the collective-level traits of each colony to the distribution of the individual-level traits from all of the measured individuals from the corresponding colony.

```
MT01<-subset(IR_deconstruct, Colony == 'MT01') # Colony MT01: Temnothorax rudis
MT01_colony<-subset(CR_deconstruct, Colony == 'MT01')
BFLT4<-subset(IR_deconstruct, Colony == 'BFLT4')
BFLT4_colony<-subset(CR_deconstruct, Colony == 'BFLT4') # Colony BFLT4: Temnothorax obturator
PPL2<-subset(IR_deconstruct, Colony == 'PPL2')
PPL2_colony<-subset(CR_deconstruct, Colony == 'PPL2') # Colony PPL2: Leptothorax crassipilis

sum(MT01$cf_wavelet>MT01_colony$cf_wavelet) / length(MT01$cf_wavelet)
```

```
## [1] 0.01538462
```

```
sum(MT01$Dominant_Period>MT01_colony$Dominant_Period) / length(MT01$Dominant_Period)
```

```
## [1] 0.2769231
```

```
sum(BFLT4$cf_wavelet>BFLT4_colony$cf_wavelet) / length(BFLT4$cf_wavelet)
```

```
## [1] 0
```

```
sum(BFLT4$Dominant_Period>BFLT4_colony$Dominant_Period) / length(BFLT4$Dominant_Period)
```

```
## [1] 0.4117647
```

```
sum(PPL2$cf_wavelet>PPL2_colony$cf_wavelet) / length(PPL2$cf_wavelet)
```

```
## [1] 0.9333333
```

```
sum(PPL2$Dominant_Period>PPL2_colony$Dominant_Period) / length(PPL2$Dominant_Period)
```

```
## [1] 0.8
```

Supplementary colony size analysis

This code section contains our supplementary analyses of the effect of colony size on the two collective-level traits. We test for correlations between the two traits and colony size for all colonies separately for four species. These four species had the greatest number of colonies in our study. We also performed PGLS analyses using all 22 species in the study. The tip-values for the PGLS were obtained using LME models.

```
CR <- read.csv("Table_S1_collective_activity.csv", header=T)

m1 <- lme(Dominant_Period ~ Species, random=~1|Colony, data=CR) # LME models for PGLS tip-valu
es.
period.CR=data.frame(summary(m1)$tTable)
period.CR$Value[2:length(period.CR$Value)]=period.CR$Value[2:length(period.CR$Value)]+period.C
R$Value[1]
```

```
row.names(period.CR)=sort(unique(CR$Species))

m1 <- lme(cf_wavelet ~ Species, random=~1|Colony, data=CR)
wavelet.CR=data.frame(summary(m1)$tTable)
wavelet.CR$Value[2:length(wavelet.CR$Value)]=wavelet.CR$Value[2:length(wavelet.CR$Value)]+wave
let.CR$Value[1]
row.names(wavelet.CR)=sort(unique(CR$Species))

m1 <- lme(No_of_ants ~ Species, random=~1|Colony, data=CR)
anova(m1)
```

```
##             numDF denDF  F-value p-value
## (Intercept)     1   292 575.1074  <.0001
## Species        21   242   4.9984  <.0001
```

```
size.CR=data.frame(summary(m1)$tTable)
size.CR$Value[2:length(size.CR$Value)]=size.CR$Value[2:length(size.CR$Value)]+size.CR$Value[1]
row.names(size.CR)=sort(unique(CR$Species))


wavelet.CR.value=setNames(wavelet.CR$Value,row.names(wavelet.CR))
period.CR.value=setNames(period.CR$Value,row.names(period.CR))
size.CR.value=setNames(size.CR$Value,row.names(size.CR))

CR3<-data.frame(period.CR.value,wavelet.CR.value, size.CR.value)
colnames(CR3)<-c("Dominant_Period","cf_wavelet", "No_of_ants")
CR3$Species<-rownames(CR3)


trudis<-subset(CR, Species == "Temnothorax_rudis") # detailed four-species analysis
tcurvispinosus<-subset(CR, Species == "Temnothorax_curvispinosus")
trugatulus<-subset(CR, Species == "Temnothorax_rugatulus")
tambiguus<-subset(CR, Species == "Temnothorax_ambiguus")

m1 <- lme(Dominant_Period ~ No_of_ants, random=~1|Colony, data=trudis)
m2 <- lme(Dominant_Period ~ No_of_ants, random=~1|Colony, data=tcurvispinosus)
m3 <- lme(Dominant_Period ~ No_of_ants, random=~1|Colony, data=trugatulus)
m4 <- lme(Dominant_Period ~ No_of_ants, random=~1|Colony, data=tambiguus)

summary(m1)
```

```
## Linear mixed-effects model fit by REML
##  Data: trudis
##        AIC      BIC    logLik
##   554.8382 563.5968 -273.4191
##
## Random effects:
##  Formula: ~1 | Colony
##         (Intercept) Residual
## StdDev:    2.931824 13.06649
##
## Fixed effects: Dominant_Period ~ No_of_ants
```

```
##                 Value Std.Error DF   t-value p-value
## (Intercept) 55.80985  3.318068 34 16.819982  0.0000
## No_of_ants   0.03410  0.020482 34  1.665027  0.1051
##  Correlation: 
##            (Intr)
## No_of_ants -0.865
## 
## Standardized Within-Group Residuals:
##           Min            Q1           Med            Q3           Max 
## -1.9142508203 -0.6702946302 -0.0005461033  0.4505985473  4.3727399915 
## 
## Number of Observations: 68
## Number of Groups: 33
```

```
summary(m2)
```

```
## Linear mixed-effects model fit by REML
##  Data: tcurvispinosus 
##        AIC      BIC   logLik
##   498.4881 506.5895 -245.244
## 
## Random effects:
##  Formula: ~1 | Colony
##         (Intercept) Residual
## StdDev:    1.489476 16.63127
## 
## Fixed effects: Dominant_Period ~ No_of_ants 
##                 Value Std.Error DF  t-value p-value
## (Intercept) 38.64531  4.042614 28 9.559487  0.0000
## No_of_ants   0.02341  0.037351 28 0.626896  0.5358
##  Correlation: 
##            (Intr)
## No_of_ants -0.839
## 
## Standardized Within-Group Residuals:
##        Min         Q1        Med         Q3        Max 
## -1.2702271 -0.7348498 -0.3107572  0.3500645  2.9874901 
## 
## Number of Observations: 58
## Number of Groups: 29
```

```
summary(m3)
```

```
## Linear mixed-effects model fit by REML
##  Data: trugatulus 
##        AIC     BIC    logLik
##   587.5496 596.486 -289.7748
## 
## Random effects:
##  Formula: ~1 | Colony
##         (Intercept) Residual
```

```
## StdDev: 0.001173935 14.23403
##
## Fixed effects: Dominant_Period ~ No_of_ants
##                 Value Std.Error DF   t-value p-value
## (Intercept) 42.52075 3.0663381 37 13.866949  0.0000
## No_of_ants   0.03598 0.0214463 37  1.677902  0.1018
##  Correlation:
##            (Intr)
## No_of_ants -0.835
##
## Standardized Within-Group Residuals:
##         Min          Q1         Med          Q3         Max
## -1.62090337 -0.79245960 -0.02813526  0.44952483  2.13824883
##
## Number of Observations: 71
## Number of Groups: 33
```

```
summary(m4)
```

```
## Linear mixed-effects model fit by REML
##  Data: tambiguus
##        AIC      BIC    logLik
##   937.8459 948.4617 -464.9229
##
## Random effects:
##  Formula: ~1 | Colony
##         (Intercept) Residual
## StdDev:    12.09656 15.35073
##
## Fixed effects: Dominant_Period ~ No_of_ants
##                 Value Std.Error DF   t-value p-value
## (Intercept) 44.01008  4.317592 57 10.193200  0.0000
## No_of_ants   0.11237  0.076513 48  1.468705  0.1484
##  Correlation:
##            (Intr)
## No_of_ants -0.862
##
## Standardized Within-Group Residuals:
##        Min         Q1        Med         Q3        Max
## -1.3077379 -0.5919431 -0.2210012  0.4992572  3.0146005
##
## Number of Observations: 107
## Number of Groups: 58
```

```
m5 <- lme(cf_wavelet ~ No_of_ants, random=~1|Colony, data=trudis)
m6 <- lme(cf_wavelet ~ No_of_ants, random=~1|Colony, data=tcurvispinosus)
m7 <- lme(cf_wavelet ~ No_of_ants, random=~1|Colony, data=trugatulus)
m8 <- lme(cf_wavelet ~ No_of_ants, random=~1|Colony, data=tambiguus)

summary(m5)
```

```
## Linear mixed-effects model fit by REML
##  Data: trudis
##        AIC      BIC    logLik
##   264.5463 273.3049 -128.2731
##
## Random effects:
##  Formula: ~1 | Colony
##         (Intercept) Residual
## StdDev:   0.6016503 1.370407
##
## Fixed effects: cf_wavelet ~ No_of_ants
##                 Value Std.Error DF   t-value p-value
## (Intercept) 4.433724 0.3815606 34 11.619971  0.0000
## No_of_ants  0.001552 0.0023357 34  0.664552  0.5108
##  Correlation:
##            (Intr)
## No_of_ants -0.857
##
## Standardized Within-Group Residuals:
##        Min         Q1        Med         Q3        Max
## -1.4750075 -0.7681532 -0.2374181  0.5933072  2.2221880
##
## Number of Observations: 68
## Number of Groups: 33
```

```
summary(m6)
```

```
## Linear mixed-effects model fit by REML
##  Data: tcurvispinosus
##        AIC      BIC    logLik
##   56.33814 64.43955 -24.16907
##
## Random effects:
##  Formula: ~1 | Colony
##         (Intercept)  Residual
## StdDev: 1.24496e-05 0.3221806
##
## Fixed effects: cf_wavelet ~ No_of_ants
##                  Value  Std.Error DF  t-value p-value
## (Intercept) 2.7798565 0.07771821 28 35.76841  0.0000
## No_of_ants  0.0005132 0.00071807 28  0.71468  0.4807
##  Correlation:
##            (Intr)
## No_of_ants -0.839
##
## Standardized Within-Group Residuals:
##         Min          Q1         Med          Q3         Max
## -1.61913618 -0.81607126 -0.06127648  0.81454677  2.32861607
##
## Number of Observations: 58
## Number of Groups: 29
```

```
summary(m7)
```

```
## Linear mixed-effects model fit by REML
##  Data: trugatulus
##        AIC     BIC    logLik
##   223.0216 231.958 -107.5108
##
## Random effects:
##  Formula: ~1 | Colony
##         (Intercept)  Residual
## StdDev:   0.2520586 0.9846191
##
## Fixed effects: cf_wavelet ~ No_of_ants
##                Value  Std.Error DF   t-value p-value
## (Intercept) 3.863102 0.22485319 37 17.180552   0.000
## No_of_ants  0.000082 0.00157713 37  0.051709   0.959
##  Correlation:
##            (Intr)
## No_of_ants -0.831
##
## Standardized Within-Group Residuals:
##        Min         Q1        Med         Q3        Max
## -1.1987956 -0.6010299 -0.2010545  0.2354593  5.1783370
##
## Number of Observations: 71
## Number of Groups: 33
```

```
summary(m8)
```

```
## Linear mixed-effects model fit by REML
##  Data: tambiguus
##        AIC      BIC    logLik
##   109.7919 120.4077 -50.89594
##
## Random effects:
##  Formula: ~1 | Colony
##         (Intercept)  Residual
## StdDev:   0.1257743 0.3432728
##
## Fixed effects: cf_wavelet ~ No_of_ants
##                  Value  Std.Error DF  t-value p-value
## (Intercept)  2.8336409 0.07352986 57 38.53728  0.0000
## No_of_ants  -0.0003625 0.00131224 48 -0.27627  0.7835
##  Correlation:
##            (Intr)
## No_of_ants -0.862
##
## Standardized Within-Group Residuals:
##        Min         Q1        Med         Q3        Max
## -2.1927703 -0.5438482 -0.1091715  0.5108622  3.2322957
##
## Number of Observations: 107
```

```
## Number of Groups: 58
```

```
# PGLS analysis
pglsModel <- gls(Dominant_Period  ~ No_of_ants , correlation = corBrownian(phy = pruned, form
= ~Species), data = CR3, method = "ML")
summary(pglsModel)
```

```
## Generalized least squares fit by maximum likelihood
##   Model: Dominant_Period ~ No_of_ants
##   Data: CR3
##        AIC      BIC    logLik
##   180.0527 183.3258 -87.02636
##
## Correlation Structure: corBrownian
##  Formula: ~Species
##  Parameter estimate(s):
## numeric(0)
##
## Coefficients:
##                Value Std.Error  t-value p-value
## (Intercept) 35.0322 19.501046 1.796426  0.0875
## No_of_ants   0.0731  0.059377 1.231178  0.2325
##
##  Correlation:
##            (Intr)
## No_of_ants -0.193
##
## Standardized residuals:
##         Min          Q1         Med          Q3         Max
## -0.67371019 -0.32547498 -0.03443393  0.33222748  0.99302336
##
## Residual standard error: 29.87395
## Degrees of freedom: 22 total; 20 residual
```

```
pglsModel <- gls(cf_wavelet ~ No_of_ants , correlation = corBrownian(phy = pruned, form = ~Spe
cies), data = CR3, method = "ML")
summary(pglsModel)
```

```
## Generalized least squares fit by maximum likelihood
##   Model: cf_wavelet ~ No_of_ants
##   Data: CR3
##        AIC      BIC    logLik
##   43.46128 46.73441 -18.73064
##
## Correlation Structure: corBrownian
##  Formula: ~Species
##  Parameter estimate(s):
## numeric(0)
##
## Coefficients:
##                  Value Std.Error  t-value p-value
```

```
## (Intercept) 2.6916044 0.8746927 3.077200  0.0059
## No_of_ants  0.0050717 0.0026633 1.904341  0.0714
##
##  Correlation:
##            (Intr)
## No_of_ants -0.193
##
## Standardized residuals:
##        Min         Q1        Med         Q3        Max
## -0.2390348 -0.1070423  0.1464364  0.3688661  0.9328575
##
## Residual standard error: 1.339955
## Degrees of freedom: 22 total; 20 residual
```

Small-nest analyses

This code section contains our analyses of our small-nest experiment. We carry out the same analyses that were performed on the dataset from the main experiment: 1. Comparing rate estimates between colony-level and individual-level traits, 2. Comparing the phenotypic disparity between the multivariate colony-level and individual-level phenospaces, and 3. Conducting PGLS regression on analogous colony-level and individual-level trait pairs.

```
# Rate analysis

# The full bootstrapping code below has been commented out since the saved output of this code
 can be loaded instead

# CR <- read.csv("Table_S1_collective_activity.csv", header=T)
#
# Tiny_2024 <- read.csv("Table_S12_Tiny_2024.csv", header=T)
#
# #Tiny_2024 <- subset(Tiny_2024, Treatment == "No_larvae")
# Tiny_2024 <- subset(Tiny_2024, Treatment == "Larvae")
#
#
# CR9 <- CR[CR$Species %in% unique(Tiny_2024$Species), ]
#
# CR9$Dominant_Period<-CR9$Dominant_Period_trim_10hrs
# CR9$cf_wavelet<-CR9$cf_wavelet_trim_10hrs
#
# df2<-NULL
# set.seed(9)
# for (i in 1:10000){
#
#   CR<-as.data.frame(CR9)
#   IR<-as.data.frame(Tiny_2024)
#   CR.sample<-sample_n(CR, nrow(CR), replace = TRUE)
#   CR.sample<-as.data.frame(CR.sample)
#   IR.sample<-sample_n(IR, nrow(IR), replace = TRUE)
#   IR.sample<-as.data.frame(IR.sample)
#   m.period.CR <- lme(Dominant_Period ~ Species, random=~1|Colony, data=CR.sample)
#   m.wavelet.CR <- lme(cf_wavelet ~ Species, random=~1|Colony, data=CR.sample)
#   m.period.IR <- lm(Dominant_Period ~ Species, data=IR.sample)
```

```
#   m.wavelet.IR <- lm(cf_wavelet ~ Species, data=IR.sample)
#
#   period.CR=data.frame(summary(m.period.CR)$tTable)
#   period.CR$Value[2:length(period.CR$Value)]=period.CR$Value[2:length(period.CR$Value)]+peri
od.CR$Value[1]
#   row.names(period.CR)=sort(unique(CR.sample$Species))
#
#   wavelet.CR=data.frame(summary(m.wavelet.CR)$tTable)
#   wavelet.CR$Value[2:length(wavelet.CR$Value)]=wavelet.CR$Value[2:length(wavelet.CR$Value)]+
wavelet.CR$Value[1]
#   row.names(wavelet.CR)=sort(unique(CR.sample$Species))
#
#   period.IR=data.frame(summary(m.period.IR)$coefficients)
#   period.IR$Estimate[2:length(period.IR$Estimate)]=period.IR$Estimate[2:length(period.IR$Est
imate)]+period.IR$Estimate[1]
#   row.names(period.IR)=sort(unique(IR.sample$Species))
#
#   wavelet.IR=data.frame(summary(m.wavelet.IR)$coefficients)
#   wavelet.IR$Estimate[2:length(wavelet.IR$Estimate)]=wavelet.IR$Estimate[2:length(wavelet.IR
$Estimate)]+wavelet.IR$Estimate[1]
#   row.names(wavelet.IR)=sort(unique(IR.sample$Species))
#
#
#
#
#   wavelet.CR.value=setNames(wavelet.CR$Value,row.names(wavelet.CR))/mean(wavelet.CR$Value)
#   wavelet.IR.value=setNames(wavelet.IR$Estimate,row.names(wavelet.IR))/mean(wavelet.IR$Estim
ate)
#   period.CR.value=setNames(period.CR$Value,row.names(period.CR))/mean(period.CR$Value)
#   period.IR.value=setNames(period.IR$Estimate,row.names(period.IR))/mean(period.IR$Estimate)
#
#
#   nc_p<-as.data.frame(unique(CR.sample$Species))
#   row.names(nc_p)<-nc_p$`unique(CR.sample$Species)`
#   nc <- name.check(t, nc_p)
#   pruned.CR <- drop.tip(t, nc$tree_not_data)
#   pruned.CR<-force.ultrametric(pruned.CR)
#
#
#   nc_p<-as.data.frame(unique(IR.sample$Species))
#   row.names(nc_p)<-nc_p$`unique(IR.sample$Species)`
#   nc <- name.check(t, nc_p)
#   pruned.IR <- drop.tip(t, nc$tree_not_data)
#   pruned.RR<-force.ultrametric(pruned.IR)
#
#
#
#   fc.wavelet.CR=fitContinuous(pruned.CR,dat=wavelet.CR.value,model="BM")
#   fc.wavelet.CR$opt
#
#   fc.wavelet.IR=fitContinuous(pruned.IR,dat=wavelet.IR.value,model="BM")
#   fc.wavelet.IR$opt
#
#   fc.period.CR=fitContinuous(pruned.CR,dat=period.CR.value,model="BM")
```

```
#   fc.period.CR$opt
#
#   fc.period.IR=fitContinuous(pruned.IR,dat=period.IR.value,model="BM")
#   fc.period.IR$opt
#
#
#   df3<-data.frame(fc.wavelet.CR$opt[1],fc.wavelet.IR$opt[1],fc.period.CR$opt[1],fc.period.IR
$opt[1])
#   colnames(df3)<-c("sigsq.wavelet.CR","sigsq.wavelet.IR", "sigsq.period.CR", "sigsq.period.I
R")
#   df3<-t(df3)
#   df3<-data.frame(df3)
#   colnames(df3)<-c("sigsq")
#   df3$sigsq.tier<-rownames(df3)
#   df3$source<-c("Colony", "Individual","Colony", "Individual")
#   df3$trait<-c("Rhythmicity", "Rhythmicity","Period", "Period")
#
#   df2<-rbind(df2,df3)
#
# }

df2<-read.csv("rates_resample_small_nest_larvae.csv", header = TRUE) # load previously complet
ed bootstrap rate data for the larvae treatment

x<-subset(df2, sigsq.tier == "sigsq.wavelet.CR")
x2<-subset(df2, sigsq.tier == "sigsq.wavelet.IR")
wavelet_r_diff<-x$sigsq-x2$sigsq # compute differences in the evolutionary rates of collective
-level and individual-level rhythmicity from the larvae treatment data
x<-subset(df2, sigsq.tier == "sigsq.period.CR")
x2<-subset(df2, sigsq.tier == "sigsq.period.IR")
period_r_diff<-x$sigsq-x2$sigsq # compute differences in the evolutionary rates of collective-
level and individual-level period from the larvae treatment data

sum(0>period_r_diff) / length(period_r_diff) # compare observed rate differences against the n
ull expectation of a difference of 0.
```

```
## [1] 0.0963
```

```
sum(0>wavelet_r_diff) / length(wavelet_r_diff)
```

```
## [1] 0
```

```
df2<-read.csv("rates_resample_small_nest_no_larvae.csv", header = TRUE)

x<-subset(df2, sigsq.tier == "sigsq.wavelet.CR")
x2<-subset(df2, sigsq.tier == "sigsq.wavelet.IR")
wavelet_r_diff<-x$sigsq-x2$sigsq # compute differences in the evolutionary rates of collective
-level and individual-level rhythmicity from the no larvae treatment data
x<-subset(df2, sigsq.tier == "sigsq.period.CR")
x2<-subset(df2, sigsq.tier == "sigsq.period.IR")
period_r_diff<-x$sigsq-x2$sigsq # compute differences in the evolutionary rates of collective-
```

```
level and individual-level period from the no larvae treatment data

sum(0>period_r_diff) / length(period_r_diff)
```

```
## [1] 0.1033
```

```
sum(0>wavelet_r_diff) / length(wavelet_r_diff)
```

```
## [1] 0
```

```
# Disparity analysis

CR_2024 <- read.csv("Table_S11_collective_activity_2024.csv", header=T)
CR.2024.values<-select(CR_2024, Dominant_Period, cf_wavelet)
rownames(CR.2024.values)<-CR_2024$Species

Tiny_2024 <- read.csv("Table_S12_Tiny_2024.csv", header=T)

Tiny_2024 <- subset(Tiny_2024, Treatment == "No_larvae")

m1 <- lm(Dominant_Period ~ Species, data=Tiny_2024)
period.Tiny=data.frame(summary(m1)$coefficients)
period.Tiny$Estimate[2:length(period.Tiny$Estimate)]=period.Tiny$Estimate[2:length(period.Tiny
$Estimate)]+period.Tiny$Estimate[1]
row.names(period.Tiny)=sort(unique(Tiny_2024$Species))
m1 <- lm(cf_wavelet ~ Species, data=Tiny_2024)
wavelet.Tiny=data.frame(summary(m1)$coefficients)
wavelet.Tiny$Estimate[2:length(wavelet.Tiny$Estimate)]=wavelet.Tiny$Estimate[2:length(wavelet.
Tiny$Estimate)]+wavelet.Tiny$Estimate[1]
row.names(wavelet.Tiny)=sort(unique(Tiny_2024$Species))
wavelet.Tiny.value=setNames(wavelet.Tiny$Estimate,row.names(wavelet.Tiny))
period.Tiny.value=setNames(period.Tiny$Estimate,row.names(period.Tiny))
IR_Tiny<-data.frame(period.Tiny.value,wavelet.Tiny.value)
colnames(IR_Tiny)<-c("Dominant_Period","cf_wavelet")


Tiny_2024 <- read.csv("Table_S12_Tiny_2024.csv", header=T)

Tiny_2024 <- subset(Tiny_2024, Treatment == "Larvae")

m1 <- lm(Dominant_Period ~ Species, data=Tiny_2024)
period.Tiny=data.frame(summary(m1)$coefficients)
period.Tiny$Estimate[2:length(period.Tiny$Estimate)]=period.Tiny$Estimate[2:length(period.Tiny
$Estimate)]+period.Tiny$Estimate[1]
row.names(period.Tiny)=sort(unique(Tiny_2024$Species))
m1 <- lm(cf_wavelet ~ Species, data=Tiny_2024)
wavelet.Tiny=data.frame(summary(m1)$coefficients)
wavelet.Tiny$Estimate[2:length(wavelet.Tiny$Estimate)]=wavelet.Tiny$Estimate[2:length(wavelet.
Tiny$Estimate)]+wavelet.Tiny$Estimate[1]
row.names(wavelet.Tiny)=sort(unique(Tiny_2024$Species))
wavelet.Tiny.value=setNames(wavelet.Tiny$Estimate,row.names(wavelet.Tiny))
```

```
period.Tiny.value=setNames(period.Tiny$Estimate,row.names(period.Tiny))
IR_Tiny_Larvae<-data.frame(period.Tiny.value,wavelet.Tiny.value)
colnames(IR_Tiny_Larvae)<-c("Dominant_Period","cf_wavelet")

combined<-combine(CR.2024.values,IR_Tiny,IR_Tiny_Larvae)

X<-subset(combined, select = -c(source))

tier<-custom.subsets(X, list(Colony = c(1:9), Individual = c(19:27))) # Larvae treatment
set.seed(9)
tier_bootstrap<-boot.matrix(tier,bootstrap=10000) # acquire bootstrap dataset using the dispRi
ty package

tier_disparity<-dispRity(tier_bootstrap,metric=c(sum,variances)) # compute disparity metric fo
r collective-level and individual-level behavior in the bootstrapped dataset

ind_boot_disparity<-as.data.frame(tier_disparity$disparity$Individual[2])
ind_boot_disparity<-t(ind_boot_disparity)
colony_boot_disparity<-as.data.frame(tier_disparity$disparity$Colony[2])
colony_boot_disparity<-t(colony_boot_disparity)
ind_boot_disparity<-as.data.frame(ind_boot_disparity)
colony_boot_disparity<-as.data.frame(colony_boot_disparity)

Individual<-ind_boot_disparity$V1
Colony<-colony_boot_disparity$V1
boot_disparity<-combine(Colony,Individual)
boot_disparity$source = paste0(boot_disparity$source,"-level")

colnames(boot_disparity)[1]<-c("Sum_of_variances")

disparity_diff<-boot_disparity$Sum_of_variances[1:10000]-boot_disparity$Sum_of_variances[10001
:20000] # compute differences in disparity from the bootstrapped data

sum(0>disparity_diff) / length(disparity_diff) # compare observed differences from the Larvae
treatment against the null expectation of a difference of 0.
```

```
## [1] 0
```

```
tier<-custom.subsets(X, list(Colony = c(1:9), Individual = c(10:18))) # No-larvae treatment
set.seed(9)
tier_bootstrap<-boot.matrix(tier,bootstrap=10000) # acquire bootstrap dataset using the dispRi
ty package

tier_disparity<-dispRity(tier_bootstrap,metric=c(sum,variances))# compute disparity metric for
 collective-level and individual-level behavior in the bootstrapped dataset

ind_boot_disparity<-as.data.frame(tier_disparity$disparity$Individual[2])
ind_boot_disparity<-t(ind_boot_disparity)
colony_boot_disparity<-as.data.frame(tier_disparity$disparity$Colony[2])
colony_boot_disparity<-t(colony_boot_disparity)
ind_boot_disparity<-as.data.frame(ind_boot_disparity)
colony_boot_disparity<-as.data.frame(colony_boot_disparity)
```

```
Individual<-ind_boot_disparity$V1
Colony<-colony_boot_disparity$V1
boot_disparity<-combine(Colony,Individual)
boot_disparity$source = paste0(boot_disparity$source,"-level")

colnames(boot_disparity)[1]<-c("Sum_of_variances")

disparity_diff<-boot_disparity$Sum_of_variances[1:10000]-boot_disparity$Sum_of_variances[10001
:20000] # compute differences in disparity from the bootstrapped data

sum(0>disparity_diff) / length(disparity_diff) # compare observed differences from the no-larv
ae treatment against the null expectation of a difference of 0.
```

```
## [1] 0
```

```
# PGLS analyses

Tiny_2024 <- read.csv("Table_S12_Tiny_2024.csv", header=T)
Tiny_2024 <- subset(Tiny_2024, Treatment == "Larvae")

m1 <- lm(Dominant_Period ~ Species, data=Tiny_2024)

period.Tiny=data.frame(summary(m1)$coefficients)
period.Tiny$Estimate[2:length(period.Tiny$Estimate)]=period.Tiny$Estimate[2:length(period.Tiny
$Estimate)]+period.Tiny$Estimate[1]
row.names(period.Tiny)=sort(unique(Tiny_2024$Species))

m1 <- lm(cf_wavelet ~ Species, data=Tiny_2024)

wavelet.Tiny=data.frame(summary(m1)$coefficients)
wavelet.Tiny$Estimate[2:length(wavelet.Tiny$Estimate)]=wavelet.Tiny$Estimate[2:length(wavelet.
Tiny$Estimate)]+wavelet.Tiny$Estimate[1]
row.names(wavelet.Tiny)=sort(unique(Tiny_2024$Species))

wavelet.Tiny.value=setNames(wavelet.Tiny$Estimate,row.names(wavelet.Tiny))
period.Tiny.value=setNames(period.Tiny$Estimate,row.names(period.Tiny))
IR_Tiny<-data.frame(period.Tiny.value,wavelet.Tiny.value)
colnames(IR_Tiny)<-c("Dominant_Period","cf_wavelet")

merged <- merge(CR.2024.values, IR_Tiny, by = 'row.names', all = FALSE)

colnames(merged)<-c("Species","Dominant_Period_2024","cf_wavelet_2024","Dominant_Period_Tiny",
"cf_wavelet_Tiny")
rownames(merged)<-merged$Species

nc <- name.check(t, merged)
pruned.Tiny <- drop.tip(t, nc$tree_not_data)
pruned.Tiny<-force.ultrametric(pruned.Tiny)

pglsModel <- gls(Dominant_Period_2024 ~ Dominant_Period_Tiny  , correlation = corBrownian(phy
= pruned.Tiny, form = ~Species), data = merged, method = "ML")
summary(pglsModel) # PGLS analysis for period - larvae treatment
```

```
## Generalized least squares fit by maximum likelihood
##   Model: Dominant_Period_2024 ~ Dominant_Period_Tiny
##   Data: merged
##        AIC      BIC    logLik
##   83.75894 84.35061 -38.87947
##
## Correlation Structure: corBrownian
##  Formula: ~Species
##  Parameter estimate(s):
## numeric(0)
##
## Coefficients:
##                          Value Std.Error    t-value p-value
## (Intercept)          -3.570187  36.67354 -0.0973505  0.9252
## Dominant_Period_Tiny  1.648754   1.07017  1.5406537  0.1673
##
##  Correlation:
##                      (Intr)
## Dominant_Period_Tiny -0.837
##
## Standardized residuals:
##         Min          Q1         Med          Q3         Max
## -1.01203198 -0.82825373  0.05095481  0.48380807  1.31893220
##
## Residual standard error: 28.02485
## Degrees of freedom: 9 total; 7 residual
```

```
pglsModel <- gls(cf_wavelet_2024 ~ cf_wavelet_Tiny  , correlation = corBrownian(phy = pruned.T
iny, form = ~Species), data = merged, method = "ML")
summary(pglsModel) # PGLS analysis for rhythmicity - larvae treatment
```

```
## Generalized least squares fit by maximum likelihood
##   Model: cf_wavelet_2024 ~ cf_wavelet_Tiny
##   Data: merged
##       AIC      BIC   logLik
##   34.8084 35.40007 -14.4042
##
## Correlation Structure: corBrownian
##  Formula: ~Species
##  Parameter estimate(s):
## numeric(0)
##
## Coefficients:
##                     Value Std.Error    t-value p-value
## (Intercept)     -3.850996  9.584739 -0.4017841  0.6998
## cf_wavelet_Tiny  2.699409  3.594852  0.7509098  0.4772
##
##  Correlation:
##                 (Intr)
## cf_wavelet_Tiny -0.99
##
```

```
## Standardized residuals:
##           Min            Q1           Med            Q3           Max
## -0.2866260446 -0.2677986038 -0.1481254214  0.0006160042  1.5614081005
##
## Residual standard error: 1.847101
## Degrees of freedom: 9 total; 7 residual
```

```
Tiny_2024 <- read.csv("Table_S12_Tiny_2024.csv", header=T)
Tiny_2024 <- subset(Tiny_2024, Treatment == "No_larvae")

m1 <- lm(Dominant_Period ~ Species, data=Tiny_2024)

period.Tiny=data.frame(summary(m1)$coefficients)
period.Tiny$Estimate[2:length(period.Tiny$Estimate)]=period.Tiny$Estimate[2:length(period.Tiny
$Estimate)]+period.Tiny$Estimate[1]
row.names(period.Tiny)=sort(unique(Tiny_2024$Species))

m1 <- lm(cf_wavelet ~ Species, data=Tiny_2024)

wavelet.Tiny=data.frame(summary(m1)$coefficients)
wavelet.Tiny$Estimate[2:length(wavelet.Tiny$Estimate)]=wavelet.Tiny$Estimate[2:length(wavelet.
Tiny$Estimate)]+wavelet.Tiny$Estimate[1]
row.names(wavelet.Tiny)=sort(unique(Tiny_2024$Species))

wavelet.Tiny.value=setNames(wavelet.Tiny$Estimate,row.names(wavelet.Tiny))
period.Tiny.value=setNames(period.Tiny$Estimate,row.names(period.Tiny))
IR_Tiny<-data.frame(period.Tiny.value,wavelet.Tiny.value)
colnames(IR_Tiny)<-c("Dominant_Period","cf_wavelet")

merged <- merge(CR.2024.values, IR_Tiny, by = 'row.names', all = FALSE)

colnames(merged)<-c("Species","Dominant_Period_2024","cf_wavelet_2024","Dominant_Period_Tiny",
"cf_wavelet_Tiny")
rownames(merged)<-merged$Species

nc <- name.check(t, merged)
pruned.Tiny <- drop.tip(t, nc$tree_not_data)
pruned.Tiny<-force.ultrametric(pruned.Tiny)

pglsModel <- gls(Dominant_Period_2024 ~ Dominant_Period_Tiny  , correlation = corBrownian(phy
= pruned.Tiny, form = ~Species), data = merged, method = "ML")
summary(pglsModel) # PGLS analysis for period - no-larvae treatment
```

```
## Generalized least squares fit by maximum likelihood
##   Model: Dominant_Period_2024 ~ Dominant_Period_Tiny
##   Data: merged
##        AIC      BIC    logLik
##   86.36751 86.95918 -40.18375
##
## Correlation Structure: corBrownian
##  Formula: ~Species
##  Parameter estimate(s):
## numeric(0)
```

```
##
## Coefficients:
##                          Value Std.Error    t-value p-value
## (Intercept)           48.52090  45.59315  1.0642147  0.3226
## Dominant_Period_Tiny -0.16509   1.34587 -0.1226674  0.9058
##
##  Correlation:
##                       (Intr)
## Dominant_Period_Tiny -0.861
##
## Standardized residuals:
##        Min         Q1        Med         Q3        Max
## -0.8613809 -0.3417638 -0.1487522  0.6646149  1.2490070
##
## Residual standard error: 32.39526
## Degrees of freedom: 9 total; 7 residual
```

```
pglsModel <- gls(cf_wavelet_2024 ~ cf_wavelet_Tiny  , correlation = corBrownian(phy = pruned.T
iny, form = ~Species), data = merged, method = "ML")
summary(pglsModel) # PGLS analysis for rhythmicity - no-larvae treatment
```

```
## Generalized least squares fit by maximum likelihood
##   Model: cf_wavelet_2024 ~ cf_wavelet_Tiny
##   Data: merged
##       AIC      BIC    logLik
##   34.7665 35.35818 -14.38325
##
## Correlation Structure: corBrownian
##  Formula: ~Species
##  Parameter estimate(s):
## numeric(0)
##
## Coefficients:
##                      Value Std.Error    t-value p-value
## (Intercept)      -3.231418  8.511701 -0.3796442  0.7155
## cf_wavelet_Tiny  2.501624  3.231849  0.7740535  0.4642
##
##  Correlation:
##                  (Intr)
## cf_wavelet_Tiny -0.988
##
## Standardized residuals:
##         Min          Q1         Med          Q3         Max
## -0.38563478 -0.15749765 -0.13189295 -0.01646072  1.60035571
##
## Residual standard error: 1.842806
## Degrees of freedom: 9 total; 7 residual
```

Locality analysis

This code section contains our supplementary analyses that shows how species collected from the same locality can differ

significantly in their collective rhythm traits.

```
CR <- read.csv("Table_S1_collective_activity.csv", header=T)

CR <- CR[CR$Origin %in% c("UC_James_San_Jacinto_Mountain_Reserve_Riverside_County_CA"), ] # As
sess species collected from the San Jacinto Mountains

CR2 <- CR %>%
  group_by(Colony, Species) %>%
  summarise_each(funs(mean))


m1 <- lme(Dominant_Period ~ Species, random=~1|Colony, data=CR) # Test for differences in colo
ny-level period among species from the San Jacinto Mountains
anova(m1)
```

```
##             numDF denDF   F-value p-value
## (Intercept)     1    28 1021.9278  <.0001
## Species         2    20    8.8602  0.0018
```

```
m1 <- lme(cf_wavelet ~ Species, random=~1|Colony, data=CR) # Test for differences in colony-le
vel rhythmicity among species from the San Jacinto Mountains
anova(m1)
```

```
##             numDF denDF  F-value p-value
## (Intercept)     1    28 619.9982  <.0001
## Species         2    20  19.9572  <.0001
```

```
CR <- read.csv("Table_S1_collective_activity.csv", header=T)

CR <- CR[CR$Origin %in% c("Pinal_Mountains_AZ"), ] # Assess species collected from the Pinal M
ountains

CR2 <- CR %>%
  group_by(Colony, Species) %>%
  summarise_each(funs(mean))

m1 <- lme(Dominant_Period ~ Species, random=~1|Colony, data=CR) # Test for differences in colo
ny-level period among species from the Pinal Mountains
anova(m1)
```

```
##             numDF denDF  F-value p-value
## (Intercept)     1    38 329.0543  <.0001
## Species         1    30   0.0000  0.9977
```

```
m1 <- lme(cf_wavelet ~ Species, random=~1|Colony, data=CR) # Test for differences in colony-le
vel rhythmicity among species from the Pinal Mountains
anova(m1)
```

```
##              numDF denDF   F-value p-value
## (Intercept)     1    38 1694.7903  <.0001
## Species         1    30   10.4943  0.0029
```
